# Low‐risk prostate lesions: An evidence review to inform discussion on losing the “cancer” label

**DOI:** 10.1002/pros.24493

**Published:** 2023-02-22

**Authors:** Caitlin R. Semsarian, Tara Ma, Brooke Nickel, Alexandra Barratt, Murali Varma, Brett Delahunt, Jeremy Millar, Lisa Parker, Paul Glasziou, Katy J. L. Bell

**Affiliations:** ^1^ Sydney School of Public Health, Faculty of Medicine and Health The University of Sydney Sydney Australia; ^2^ Department of Cellular Pathology University Hospital of Wales Cardiff UK; ^3^ Wellington School of Medicine and Health Sciences University of Otago Wellington New Zealand; ^4^ Alfred Health Radiation Oncology, The Alfred Melbourne Australia; ^5^ Charles Perkins Centre, Sydney School of Pharmacy, Faculty of Medicine and Health The University of Sydney Sydney Australia; ^6^ Department of Radiation Oncology Royal North Shore Hospital St Leonards Australia; ^7^ Institute for Evidence‐Based Healthcare, Faculty of Health Sciences and Medicine Bond University Gold Coast Australia

**Keywords:** classification, diagnosis, pathology, prostate, terminology, treatment

## Abstract

**Background:**

Active surveillance (AS) mitigates harms from overtreatment of low‐risk prostate lesions. Recalibration of diagnostic thresholds to redefine which prostate lesions are considered “cancer” and/or adopting alternative diagnostic labels could increase AS uptake and continuation.

**Methods:**

We searched PubMed and EMBASE to October 2021 for evidence on: (1) clinical outcomes of AS, (2) subclinical prostate cancer at autopsy, (3) reproducibility of histopathological diagnosis, and (4) diagnostic drift. Evidence is presented via narrative synthesis.

**Results:**

AS: one systematic review (13 studies) of men undergoing AS found that prostate cancer‐specific mortality was 0%−6% at 15 years. There was eventual termination of AS and conversion to treatment in 45%−66% of men. Four additional cohort studies reported very low rates of metastasis (0%−2.1%) and prostate cancer‐specific mortality (0%−0.1%) over follow‐up to 15 years. Overall, AS was terminated without medical indication in 1%−9% of men. Subclinical reservoir: 1 systematic review (29 studies) estimated that the subclinical cancer prevalence was 5% at <30 years, and increased nonlinearly to 59% by >79 years. Four additional autopsy studies (mean age: 54−72 years) reported prevalences of 12%−43%. Reproducibility: 1 recent well‐conducted study found high reproducibility for low‐risk prostate cancer diagnosis, but this was more variable in 7 other studies. Diagnostic drift: 4 studies provided consistent evidence of diagnostic drift, with the most recent (published 2020) reporting that 66% of cases were upgraded and 3% were downgraded when using contemporary diagnostic criteria compared to original diagnoses (1985−1995).

**Conclusions:**

Evidence collated may inform discussion of diagnostic changes for low‐risk prostate lesions.

## 1 INTRODUCTION

In higher income countries including the USA,^1^ Australia,^2^ and Canada,^3^ a dramatic increase in the incidence of prostate cancer diagnoses occurred in the late 1980s and 1990s with the widespread uptake of prostate‐specific antigen (PSA) testing.^4^ Much of the increase was driven by the detection of low‐risk prostate cancers with Gleason scores (GS) ≤6 on histopathology.^5^ Subsequently, prostate cancer incidence has remained high. Although smaller in absolute terms, there have also been decreases in rates of first diagnosis at an advanced stage, and in prostate cancer mortality.^4^, ^6^ By identifying aggressive lesions at an early stage, PSA screening may have prevented some men from morbidity associated with advanced stage cancer (and its treatment), and some from dying of prostate cancer.^6^ Against these potential benefits, harms from PSA screening include false‐positive and false‐negative results, biopsy complications (e.g., infection following transrectal biopsy, erectile dysfunction following transperineal biopsy), overdiagnosis,^7^ and overtreatment^8^, ^9^, ^10^ with risk of adverse outcomes (e.g., postoperative complications, erectile dysfunction, and urinary incontinence).^11^, ^12^

To prevent harms from overtreatment, active surveillance has emerged as a preferred management option for low‐risk lesions.^13^ The use of active surveillance for low‐risk lesions has increased in recent years, but there remains substantial variation in uptake between countries^14^, ^15^, ^16^, ^17^ (ranging from 50% in United States of America^14^ to >90% in England and Wales^17^), and within countries, with lower uptake noted among minoritized racial groups^18^ and those outside metropolitan areas.^15^, ^18^ Clinicians may not recommend active surveillance to patients because of a patient's clinical and personal characteristics (e.g., younger age), and perceptions of: patient disinterest, inadequacy of biopsy sampling, inconsistency in active surveillance guidelines, and inability of some patients to adhere to follow‐up protocols.^19^, ^20^ While a clinician's recommendation is the most important factor in influencing a patient's decision to undergo active surveillance,^21^, ^22^ patients may also be unaware of conservative management options, or have difficulty understanding and weighing up treatment options.^22^, ^23^, ^24^, ^25^ The thought of not removing the cancer may also provoke fear of cancer growth and metastasis, which may contribute to decisions to opt out from active surveillance once started.^13^

Given these significant patient and clinician barriers, effective strategies are needed to promote acceptance of active surveillance for men with low‐risk lesions. Two possible strategies targeting the pathology diagnosis are (i) the recalibration of diagnostic thresholds, whereby the criteria by which prostate lesions are considered to be “cancer” is narrowed to only those at higher risk of progression^26^, ^27^ and/or (ii) adopting alternative diagnostic labels to describe low‐risk prostate cancer lesions.^7^, ^28^, ^29^ There is evidence that relabeling other low‐risk lesions such as thyroid papillary microcarcinoma and ductal carcinoma in situ of the breast without using the word “cancer” may increase uptake of active surveillance and other conservative management options.^30^, ^31^, ^32^ In the context of prostate cancer, the recent adoption of the International Society of Urological Pathology (ISUP) Grade Group system in pathology diagnosis was patient‐centric and may reduce overtreatment of low‐risk lesions. Relabeling “GS6” lesions as “GG1” lesions emphasizes the low‐grade nature of the lesion to patients, having been assigned the most benign classification in the Grade Group system comprising grades 1−5, which may increase acceptability for conservative management options.^33^, ^34^

To inform consideration of potential changes to pathology diagnosis, we aimed to systematically search and synthesize published evidence to support or reject a change in the diagnostic criteria and/or terminology used to describe low‐risk prostate lesions. Specifically, we sought to answer four research questions about low‐risk prostatic lesions (GS6/GG1): (1) What are the clinical outcomes from active surveillance (compared to immediate treatment)? (2) What is the size of the reservoir of subclinical prostate cancer in men who died from other causes? (3) What is the reproducibility of the histopathological diagnosis? and (4) Is there evidence of diagnostic drift in the histopathological threshold (i.e., a shift over time in the diagnostic label given to the same lesion)?

## 2 METHODS

### 2.1 Data sources and search strategy

We conducted an evidence review of published original research articles, with separate searches of the EMBASE and PubMed databases to address the four research questions. All searches were limited to human studies published up to October 29, 2021, using combinations of search terms including prostate, Gleason, and their variations (search strategies in Supporting Information: 1). We also searched references and forward citations to identify additional papers.

### 2.2 Study selection

Titles and abstracts retrieved from the database searches were screened by one reviewer (C. R. S.). The full texts of potentially relevant articles were retrieved and independently screened by two reviewers (C. R. S. and T. M.), with disagreements adjudicated by a third reviewer (K. J. L. B.). Data was extracted by C. R. S. and T. L. When data were not available, we extracted data from figures using plot digitizer software (https://apps.automeris.io/wpd/, accessed June 20, 2022).

### 2.3 Inclusion criteria

We included original studies published in the English language that examined prostatic lesions that were diagnosed as GS6. For Question One (active surveillance), prospective studies that reported on relevant clinical outcomes (e.g., disease progression, mortality rates) at 5 years or beyond, who were diagnosed with low‐risk prostate cancer lesion (GS ≤6/GG ≤1 and PSA < 10 ng/mL) and not actively treated were included. For Question Two (autopsy studies), autopsy studies comprising a systematic histological examination of the prostate from adults with no known history of preexisting prostate cancer or other prostatic pathology were included. If studies examined overlapping cohorts, both studies were included as no pooling of data for meta‐analysis was required. For Question Three (reproducibility of histopathology diagnosis), studies reporting on the independent diagnostic assessments reported independently by ≥10 pathologists for the same set of histopathological slides were included. Studies needed to include diagnostic differentiation of GS6 versus GS7 lesions (3 + 4 or 4 + 3), or GG1 versus GG2 lesions, or absence versus presence of Gleason pattern (GP) 4. For Question Four (diagnostic drift), studies needed to report ≥2 independent readings of the same histopathological slides by pathologists at ≥2 time points for the purpose of diagnostic classification.

Preexisting systematic reviews were identified for both the active surveillance^13^ and autopsy^35^ questions, and provide summaries of the relevant evidence published before October 2017 and July 2013, respectively which we included in our review. Therefore, only studies that were published after these dates were additionally considered for inclusion in those categories.

### 2.4 Exclusion criteria

We excluded abstracts, protocols, review articles, and opinion articles if no new data were presented. We also excluded studies where Gleason grading was not used, and where study cohorts comprised patients with only high‐grade prostatic lesions or metastatic disease. Among the active surveillance studies, we excluded studies that did not report on clinical outcomes relevant to disease progression, retrospective studies, and studies with <100 participants. Among the autopsy studies, we excluded studies where a systematic histological examination of the prostate was not performed, and studies with <100 cases. Among the reproducibility studies, we excluded studies with <10 independent assessments of the same histopathological slides. Given the large quantity of reproducibility studies retrieved, we also excluded studies that were judged to be at high risk of bias for representativeness of the sample (extent to which included participants were representative of the target clinical population).

### 2.5 Data collection and synthesis

The data extraction templates were developed and piloted by two reviewers (B. N. and K. J. L. B). Data were extracted by one reviewer (C. R. S.) into an electronic datasheet and then reviewed by a second reviewer (T. M.) with disagreement resolved through discussion. We did not attempt to pool data, but instead undertook a narrative synthesis of the evidence in each category.

### 2.6 Quality assessment of primary studies

All studies were assessed for risk of bias by C. R. S. and K. J. L. B. using a list of standardized items adapted from the ROBINS‐I tool^36^ (active surveillance studies), the tool by Hoy et al.^37^ (autopsy studies), QUADAS‐2,^38^ and QUAREL^39^ tools (diagnostic reproducibility, diagnostic drift). For Question One (active surveillance studies), the items included baseline and/or time‐varying confounding of the effect of the intervention (i.e., active surveillance compared to immediate treatment), selection bias, classification bias, deviation from intended outcomes, nonavailability of data, measurement bias, and reporting bias. For Question Two (autopsy studies), the items included representativeness of the sample, inclusion bias, selection bias, nonavailability of data, reporting bias, application of appropriate diagnostic criteria, reliability and validity of examination method used, and consistency of cancer detection method. For Questions Three (reproducibility studies) and Four (diagnostic drift studies), the items included representativeness of the sample, blinding to previous diagnoses of specimens, diagnostic criteria application and interpretation, and statistical measures of agreement (see Supporting Information: 4 for risk of bias tools). Results of the risk of bias assessment were depicted using the online robvis tool (https://mcguinlu.shinyapps.io/robvis/).^40^

## 3 RESULTS

### 3.1 **What are the clinical outcomes from active surveillance of low‐risk prostate lesions? (*n*** = **17**, Table 1, Figure 1 **)**

**Figure 1:**
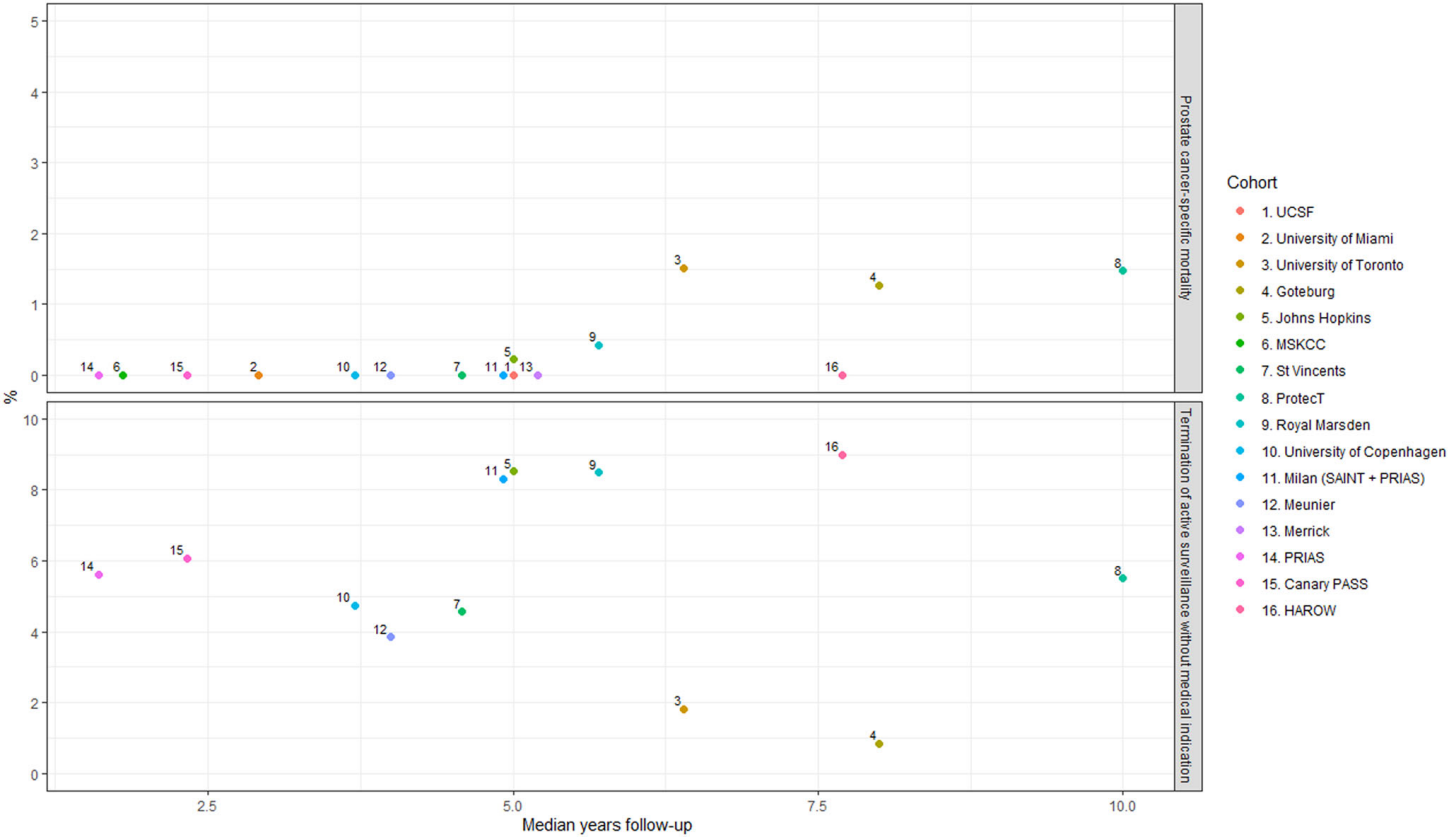
Prostate cancer‐specific mortality and termination of active surveillance without medical indication. Top panel, prostate specific mortality; Bottom panel, termination of active surveillance without medical indication. UCSF: Welty^48^, University of Miami: Soloway^55^, University of Toronto: Klotz^51^, Goteburg: Godtman^46^, Johns Hopkins: Tosoian^41^, MSKCC: Adamy^6^, St Vincents: Thompson^56^, ProtecT: Hamdy^45^, Royal Marsden: Selvadurai^50^, University of Copenhagen: Thomsen^52^, Milan: Marenghi^54^, Meunier: Meunier^42^, Merrick: Merrick^43^, PRIAS: Bul^49^, Canary Pass: Newcomb^53^, HAROW: Herden^44^. HAROW, hormonal therapy, active surveillance, radiation, operation, watchful waiting; PRIAS, prostate cancer research international active surveillance; ProtecT, prostate testing for cancer and treatment. [Color figure can be viewed at wileyonlinelibrary.com]

We screened the titles and abstracts of 247 articles identified through our database searches (246 published after the Kinsella review^13^ and 1 published in 2017 but not included in the Kinsella review^42^), resulting in 82 full texts to screen, of which 78 were excluded (flow diagram in Supporting Information: 2a, reasons for exclusion in Supporting Information: 3a). We included 4 new studies and the 13 studies from the Kinsella review^13^ in our narrative synthesis (Table 1). Prostate cancer‐specific mortality and rates of termination of active surveillance without medical indication from these studies are summarized in Figure 1.

**Table 1:** Prostate cancer active surveillance studies.

Author	Enrollment period	Country (ethnicity if reported)	Study design	Sample size	Age (years)	Lesions included	Follow‐up period	Cases of tumor progression	Incidence of metastasis	Prostate cancer‐specific mortality	Conversion to active treatment, conversion without medical indication	Overall risk of bias^a^
Kinsella^13^, b	Range: 1990−2016	USA, USA (88.4% White, 7.4% African American, 4.2% Other), USA (87% White), USA (91% Caucasian, 6% African American, 3% Asian, 1% Other), USA, UK (99% White), UK, Canada, Denmark, Australia, Sweden, Italy, Worldwide (17 countries)	Systematic review (2 RCTs, 9 prospective cohort studies, 2 retrospective cohort studies)	Range: 230−2494, total: 10,354	Median range: 62−68	GS: all include GS ≤3 + 3, 5 studies also include GS3 + 4	Median range: 8.4 months‐11 years	Does not report	Does not report	Range: 0.6%−4% at 5 years (3 studies), 0%−1.9% at 10 years (4 studies, excl. IRPC), 0%−6% at 15 years (3 studies, excl. IRPC)	Range: 25%−50% at 5 years (5 studies), 50%−53% at 10 years (2 studies), 45%−66% at 15 years (4 studies)	No ratings provided
						T score: half of studies included ≤T2a, 2 studies included T1c only, 3 studies included patients with T2b, 2 studies included patients with T2c						
											Conversion without medical indication range: 0.8%−8.5% (9 studies)	
						PSA: 10 studies used <10 μg/L, others used 15−20 or <20 μg/L						
						PSAD: included in 5 cohorts (range <0.15−0.2 ng/mL/cm^3^)						
						Positive cores: included in 11 studies (2−3 in most cases)						
						% Cancer in core: included in 8 studies (range 20%−50%)						
Tosoian^41^ (Johns Hopkins)	1995−2018	USA (87% Caucasian, 8% African American, 5% Other)	Prospective	1818 (1293 VLRPC, 525 LRPC)	VLRPC: median 66, (IQR: 61−69), LRPC: median 67, (IQR 62−71)	VLRPC: T1c, GG1, ≤2 positive biopsies with ≤50% cancer in each core, PSA < 0.15 ng/mL	For men at risk of upgrading median 5.0 years (IQR: 2.0−9.0), overall 920 followed for ≥5 years, 305 followed for ≥10 years	21%, 30%, and 32% cumulative incidences of upgrading to GG ≥2 at 5, 10, and 15 years, respectively	0.1% combined incidence of PCSM or metastasis at 10‐ and 15‐years	0.1% at 10‐ and 15‐years	36%, 48%, and 52% cumulative incidences of conversion to active treatment at 5, 10, and 15 years, respectively	High
						LRPC: ≤T2a, GG1, PSA < 10 ng/mL						
											8.5% converted to active treatment without medical indication over a median of 5 years	
Meunier^42^	2005−2016	French West Indies (African Caribbean)	Prospective	234 (190 VLRPC, 25 LRPC, 19 IRPC)	Median age at diagnosis 64 (IQR: 59−70)	VLRPC: GS ≤6 and PSA ≤10 ng/mL, ≤2 positive biopsies, and ≤3 mm per biopsy	Median 4.0 years (IQR: 2.3−5.5)	Does not report number with disease progression, 1/234 (0.4%) had lymph node involvement	0% (0/234)	0% at 2.5, 5, and 10 years	83/234 (35.5%) treated, 10/234 (4.3%) stopped AS and awaiting treatment	High
						LRPC: GS ≤6 and PSA ≤10 ng/mL					3.9% converted to active treatment without medical indication over a median of 4 years	
						IRPC: GS3 + 4 and/or PSA 10−20 ng/mL, and life expectancy <10 years with significant comorbidities						
Merrick^43^	2005−2018	USA (97.7% White, 2.1% Black, 0.3% Other)	Prospective	340 (323 GS3 + 3, 13 GS3 + 4)	Mean 65.7	T1c, GS3 + 3, and PSAD < 0.22 ng/mL/cm^3^ OR, GS3 + 4, PSA < 10 ng/mL, ≤3 positive biopsies, and ≥70 years	Median 5.2 years, (range: 1−14)	Does not report	0% (0/340)	0% (0 deaths from prostate cancer at time of reporting)	4.7%, 2.2%, 9.5%, and 25.0% for BMI cohorts <25, 25−29.9, 30−34.9, and ≥35 at 10 years, respectively (7% overall)	High
											Reasons for termination of active surveillance not reported	
Herden^44^ (HAROW)	2008−2013	Germany	Prospective	329 (207 VLRPC, 70 LRPC, 52 IRPC)	Median 69.0 (IQR: 63.4−72.5)	≤T2c, GG ≤1, ≤2 positive biopsies, PSA ≤10 ng/mL, and PSAD ≤0.2 ng/mL^2^	Median 7.7 years (IQR: 4.7−9.1)	Does not report	2.1% (7/329)	0% (0 deaths from prostate cancer at time of reporting)	187/329 (56.8%)	High
											9% converted to active treatment without medical indication over a median of 7.7 years	

Abbreviations: AS, active surveillance; GG, grade group; GS, Gleason score; HAROW, hormonal therapy, active surveillance, radiation, operation, watchful waiting; IQR, interquartile range; IRPC, intermediate risk prostate cancer; LRPC, low risk prostate cancer; MCCL, maximum cancer core length; PCSM, prostate cancer‐specific mortality; PSA, prostate‐specific antigen; PSAD, prostate‐specific antigen density; RCTs, randomized controlled trials; VLRPC, very low risk prostate cancer.

^a^ See Supporting Information: Figure S4a for complete risk of bias assessment.

^b^ Systematic review to October 2017 identifying 13 cohorts. Nil meta‐analyses performed.

The Kinsella review^13^ included two randomized controlled trials (RCTs),^45^, ^46^ 9 prospective cohort studies,^6^, ^47^, ^48^, ^49^, ^50^, ^51^, ^52^, ^53^, ^54^ and 2 retrospective cohort studies^55^, ^56^ of active surveillance, with data collected from 1990 to 2016 from 10,354 patients from cohort studies that were multinational,^49^ and in the United States of America,^6^, ^47^, ^48^, ^53^, ^55^ UK,^45^, ^50^ Canada,^51^ Denmark,^52^ Australia,^56^ Sweden,^46^ and Italy.^54^ No risk of bias assessment was reported. Prostate cancer‐specific mortality rates were low in all studies, and similar rates of conversion from active surveillance to treatment were reported. The RCTs, Prostate Testing for Cancer and Treatment (ProtecT)^45^ and Goteborg,^46^ reported 10‐year prostate cancer‐specific mortality rates of 1.2% and 0% respectively in the active surveillance arms. In the two trials 55% (ProtecT) and 66% (Goteborg) converted to treatment by 15 years. Conversion to treatment without evidence of cancer progression occurred in at least 5.5% and 0.8% of all ProtecT and Goteborg patients, respectively. The two largest prospective cohort studies reported similar findings, with Prostate Cancer Research International Active Surveillance (PRIAS)^49^ reporting 0% prostate cancer‐mortality at 4 years (median follow‐up 1.6 years). In this study, 21.1% converted to active surveillance, with 5.6% of the total cohort converting without evidence of cancer progression. Updated outcomes from the Johns Hopkins cohort are reported below. The Kinsella review also highlighted heterogeneity among active surveillance practices, including variability in inclusion criteria, monitoring protocols, and triggers for intervention.

The 4 new studies identified included 1 study reporting updated long‐term outcomes from the Johns Hopkins active surveillance cohort, and 3 new long‐term prospective cohort studies. All 4 studies were rated as at high risk of bias from potential confounding by factors associated with active surveillance and outcomes (Supporting Information: 4a). Tosoian reported long‐term outcomes of the Johns Hopkins active surveillance cohort (*n* = 1818, 1995−2018).^41^ Initially only men with very‐low risk prostate cancer (*n* = 1293) were included, but later patients with low‐risk prostate cancer (*n* = 525) were also included. After 15 years, the combined rate of metastasis (*n* = 1) and prostate cancer‐specific mortality (*n* = 4) was 0.1%. The proportion who converted to treatment (without GG ≥2 cancer on monitoring biopsy in brackets) increased over time: 36% (15%) at 5 years, 48% (18%) at 10 years, and 52% (20%) at 15‐years.^41^

Meunier described a prospective study in an African Caribbean cohort (*n* = 234, 2005−2016). Participants had very low‐risk prostate cancer (*n* = 190), low‐risk prostate cancer (*n* = 25), or favorable intermediate‐risk prostate cancer (*n* = 19). After 10 years, the rate of metastasis was 0.4% (one local lymph node metastasis) and prostate cancer‐specific mortality was 0%. After a median follow‐up of 4 years, 40% (*n* = 93) had terminated active surveillance; this was not medically indicated in 3.9% (patient preference *n* = 7, noncompliance *n* = 2).^42^

Merrick reported a prospective study in the USA cohort (*n* = 340, 2005−2018). The rate of metastasis or prostate cancer‐specific mortality was 0% over a median follow‐up of 5.2 years. By 10 years, treatment was initiated in 7% (*n* = 24), with median time to treatment of 4.9 years. Reasons for terminating active surveillance were not described.^43^

Finally, Herden reported on the prospective Hormonal therapy, Active Surveillance, Radiation, Operation, Watchful Waiting (HAROW) cohort study in Germany (*n* = 329, 2008−2019).^44^ Participants had very low‐risk (*n* = 207), low‐risk (*n* = 70), or intermediate or high‐risk prostate cancer (*n* = 52). After 10 years the risk of metastasis was 3%, and prostate cancer‐specific mortality was 0%. After a median follow‐up of 7.7 years, 57% (187/329) had terminated active surveillance and were treated, including 9% (*n* = 31) due to patient wish or physician's advice (without GG ≥2 cancer on monitoring biopsy or PSA rise).

### 3.2 **What is the size of the reservoir of subclinical prostate cancer in men not known to have prostate cancer during their lifetime? (*n*** = **33**, Table 2 **)**

We screened the titles and abstracts of 78 articles identified through our database searches, published after the July 2013 search of the Bell review,^35^ resulting in 16 full text to screen, of which 12 were excluded (flow diagram in Supporting Information: 2b, reasons for exclusion in Supporting Information: 3b). We included 4 new studies and the 29 studies from the Bell review in our narrative synthesis^35^ (Table 2).

**Table 2:** Autopsy studies of subclinical (latent) prostate cancer.

Author	Year	Country (ethnicity if applicable)	Study period	Pathologist type	Men included in study (*n*)	Age (years)	Study population	Mean pathology section width (mm)	Delay before autopsy	Prevalence (%)		Overall risk of bias^a^
										Latent prostate cancer	Latent prostate cancer with Gleason score ≤6	
Bell^35^, b	2015	USA (60% Black, 40% White), USA (59% White, 41% Black), USA (92% White, 4% Black, 0.6% Hispanic, 3% unknown), USA (64% Black, 36% White), USA (86% White, 14% Black), USA and Japan, Japan, Japan, Japan, Japan, Japan, Japanese and Russian, UK, UK, Norway, Norway, China, Sweden, Denmark, Greece, Canada, Brazil, Iran, Spain, Hungary, Turkey, Switzerland, Finland, Worldwide (seven countries)	Publication dates: 1948−2013	Does not report	Range: 100−1327, total: 8776	Varies by study	8 forensic, 19 hospital, 2 mixed	Range: 2.5–5	Varies by study	Latent prostate cancer was identified in all populations, range 3%−43%	Gleason scoring was used in 12 studies. Of these studies, the majority of prostate cancers identified were GS ≤6 in 9 studies, GS > 6 in 1 study, and not reported in 2 studies	No ratings provided
Kido^57^	2015	Japan	2002−2005	Experienced pathologist	196	Mean: 54 ± 21, range: 0−90	Forensic	Does not report	<48 h	24/196 (12.2)	10/196 (5.1)	High
Takaso^58^	2020	Japan	2004−2014	Experienced pathologist	317	Mean: 56.4 ± 17.8, range: 14−94	Forensic	Does not report	Median: 1.5 day, IQR: 1.0−2.9 day	45/317 (14.2)	17/317 (5.4)	High
Kimura^59^	2016	Japan	2008−2013	Pathologist	127	Mean: 68.9, range: 24−92	Hospital	4	<24 h	55/127 (43.3)	31/127 (24.4)	High
Inaba^60^	2020	Japan	2009−2017	Genitourinary pathologist	182	Median: 72, range: 41−97	Hospital	4	Does not report	71/182 (39.0)	43/182 (23.6)	High

*Note*: Takaso and Kido (forensic cohorts), and Inaba and Kimura (hospital cohorts) represent overlapping cohorts.

Abbreviation: IQR, interquartile range.

^a^ See Supporting Information: Figure S4b for complete risk of bias assessment.

^b^ Systematic review to July 2013 identifying 29 studies.

Bell systematically reviewed 29 autopsy studies examining the incidence of subclinical (latent) prostate cancer published before July 2013 (1948−2013). Studies described 19 hospital populations, 8 forensic populations, and 2 mixed hospital/forensic populations, including 8776 men (100−1327 per study) from 23 countries. Subclinical prostate cancer was identified in all populations, with prevalence rates ranging from 3% to 43%. There were no obvious time trends in prevalence, and age was the only significant predictive factor. The prevalence increased with each decade of age (OR: 1.7, 1.6−1.8) from 5% at age <30 years, to 59% by age >79 years.

Our search identified 4 additional autopsy studies including 2 forensic and 2 hospital cohorts. All studies were rated at a high risk of bias due to non‐representativeness of the study sample or to minimal sampling of prostate tissue (Supporting Information: 4b). The overlapping studies by Kido et al.^57^ and Takaso et al.^58^ describe forensic autopsies performed at Dokkyo Medical University, Japan from August 2002 to July 2005 (*n* = 196), and November 2004 to February 2014 (*n* = 317), respectively. In both studies, the prostate gland was cut into 3 vertical slices, and only 2 cross‐sections were examined. In the study by Kido, subclinical prostate cancer was identified in 12% (24/196) of decedents, with 42% (10/24) of the subclinical prostate cancers classified as GS ≤6. Mean (SD) age of decedents was 54 ± 21 years. Similarly, in the study by Takaso, subclinical prostate cancer was identified in 14% (45/317) of autopsies, with 38% (17/45) of cancers classified as GS ≤6. Mean (SD) age of decedents was 56 ± 18 years.

We also identified 2 new overlapping hospital autopsy studies conducted at Jikei University Hospital. The studies by Kimura et al.^59^ and Inaba et al.^60^ examined cases from 2008 to 2013, and from 2009 to 2017, respectively. In both studies, prostate glands were step‐sectioned at 4 mm intervals and examined by genitourinary pathologists. In the earlier study by Kimura, subclinical prostate cancer was identified at autopsy in 43% (55/127) of cases, with 56% (31/55) of cancers classified as GS ≤6. Mean age of decedents was 69 years (range 24−92).^59^ Inaba et al. identified subclinical prostate cancer in 39% of (71/182) cases, with 61% (43/71) of cancers classified as GS ≤6. Median age of decedents was 72 years (range 41−97).

### 3.3 What is the reproducibility of the histopathological diagnosis of Gleason score 6 prostate lesions? (*n* = 8, Table 3)

We screened the titles and abstracts of 235 articles identified through our database searches, and 2 additional articles^68^, ^70^ identified in the reference list of an included article.^67^ This resulted in 120 full texts to screen, of which 112 were excluded (flow diagram in Supporting Information: 2c, reasons for exclusion in Supporting Information: 3c). We included 8 studies in our narrative synthesis (Table 3). One study was rated at low risk of bias, and 7 studies were rated at moderate risk of bias (Supporting Information: 4c).

**Table 3:** Reproducibility studies.^a^

Author	Diagnostic categories used for analysis of diagnoses	Year	Country	Type of pathologists (*n*)	Case selection	Cases (*n*)	Key findings	Overall risk of bias^b^
Bulten^61^	Gleason grades 1−5	2020	Netherlands	Urologic pathologists (validation group: 3), pathologists (observer group: 13), pathologists in training (observer group: 2)	100 observer study images were sampled (nonrandom selection of 20 benign cases and random selection of 80 cancer cases within each Gleason grade group) from a larger pool of 550 images randomly selected from a US medical center	Validation study: 550	In the initial validation study, there was complete consensus of Gleason grades in 333/550 (61%) cases and quadratic Cohen's *ϰ* was 0.925. In the observer group, the median interrater agreement with validation consensus Gleason grade was 0.819 (quadratic *ϰ*, 95% CI: 0.726−0.869).	Low
						Observer study: 100		
Strom^62^	Gleason grades 1−5	2020	International	Urologic pathologists (23)	Images of prostate cancer biopsies from the pathology Imagebase^63^	87	Pairwise *ϰ* between observers was 0.60−0.73.	Moderate
Egevad^64^	Gleason grades 1−5	2001	Sweden	Pathologists (73), pathology residents (12)	Prostate cancer images from archival specimens selected to represent a spectrum of Gleason scores and reviewed by an expert genitourinary panel	Round 1: 20	In round 1 (before teaching), 1198/1700 (70.5%) of grades were correct, 119/1700 (7.0%) were over‐graded, and 383/1700 (22.5%) were undergraded. In round 2 (after teaching), 1472/1700 (86.6%) were correct, 69/1700 (4.1%) were over‐graded, and 159/1700 (9.4%) were undergraded.	Moderate
						Round 2: 20		
Harnden^65^	Gleason score categories 2−4, 5−6, 7, and 8−10	2008	UK	Urologic pathologists (8), pathologists (32)	Prostate biopsies selected from a pathology department selected to represent a spectrum of benign, borderline, and invasive prostate pathologies	48	Consensus (≥75% agreement) was reached in 16/18 invasive cases. Of the 5 cases with a consensus of Gleason scores 5−6, there was urologic pathologist agreement in 28/40 (70.0%) and a *ϰ* of 0.41. There was pathologist agreement in 108/131 (82.4%) and a *ϰ* of 0.49.	Moderate
Griffiths^66^	Gleason score categories 2−4, 5−6, 7, and 8−10	2006	Wales	Pathologists (24)	Prostate biopsies selected from pathologist contributions to represent a spectrum of Gleason scores, reviewed by a uropathology panel	20	The *ϰ* for interobserver agreement before teaching session was 0.27 for Gleason scores 5−6. For cases agreed to be Gleason score 5−6 according to uropathology consensus, there was 109/167 (65%) cases of pathologist agreement, 19/167 (11%) cases undergraded, and 39/167 (23%) cases over‐graded. The *ϰ* for interobserver agreement after teaching session was 0.40 for Gleason scores 5−6. There was agreement in 127/166 (77%) cases of pathologist agreement, 11/166 (7%) cases undergraded and 28/166 (17%) cases over‐graded.	Moderate
De la Taille^67^	Gleason score categories 4−6, 7, and 8−10	2003	France	Genito‐urinary pathologists (10)	Prostate cancer TMA images randomly selected from an image bank and reviewed by two expert genitourinary pathologists	537	The overall *ϰ* coefficient for interobserver agreement among 4 pathologists who evaluated 537 cases ranged from 0.45−0.69. The number of cases diagnosed as Gleason scores 4−6 ranged from 44.2%−74.4% among the 4 pathologists. The overall *ϰ* coefficient for interobserver agreement among 10 pathologists who evaluated 104 cases ranged from 0.28−0.54. The number of cases diagnosed as Gleason scores 4−6 ranged from 18.5%−68.4%.	Moderate
Lessells^68^	Gleason score categories 2−4, 5−6, and 7−10	1997	Scotland	Pathologists (12)	Prostate cancer biopsies from a pathology department	46	The *ϰ* value was 0.41 when 3 categories (2−4, 5−6, and 7−10) were used. The *ϰ* value was 0.54 when 2 categories were used (2−6 and 7−10).	Moderate
McKenney^69^	Gleason patterns 3−5	2011	USA	Genito‐urinary pathologists (11)	Images of prostate cancer biopsies from a pathology department selected to represent a spectrum of Gleason patterns 3−5	Set 1: 17	The overall *ϰ* for set 1 (classic patterns) was 0.76. The overall *ϰ* for set 2 (tangentially sectioned Gleason grade 3 or 4 with poorly formed glands) was 0.27.	Moderate
						Set 2: 17		

Abbreviations: RP, radical prostatectomy; TMA, tissue microarray; TURP, transurethral resection of prostate.

^a^ Reproducibility studies refer to cross‐sectional studies whereby there were two or more independent readings of the same histopathological slides by three or more pathologists for the purpose of diagnostic classification.

^b^ See Supporting Information: Figure S4c for complete risk of bias assessment.

The cross‐sectional reproducibility study by Bulten et al. (published in 2020)^61^ was rated at low risk of bias. This study circulated a set of 100 prostate biopsies to 13 pathologists and 2 pathologists‐in‐training from 14 independent laboratories across 10 countries (see Table 3 for case selection). Reference diagnoses were set by consensus between 3 expert uropathologists. Using diagnostic categories of GG 1−5, this study demonstrated a median interrater agreement with the consensus diagnoses (quadratic *ϰ*: 0.82, 95% CI: 0.73−0.87). In this study, a deep learning system performed at least as well as the participating pathologists (quadratic *ϰ*: 0.85, 95% CI: 0.78−0.91).

Among the 7 other reproducibility studies rated at moderate risk of bias, study design and rates of interobserver agreement varied. Studies by Strom et al.^62^ and Egevad et al.^64^ also examined diagnostic categories of GG1‐5. Strom compared Gleason grades given by 23 urologic pathologists and found moderate agreement (pairwise *ϰ* between observers ranged from 0.60 to 0.73), with a deep learning system performing similarly (pairwise *ϰ*: 0.62). Egevad compared the Gleason grades given by 73 pathologists and 12 pathology residents to consensus expert diagnoses and found that 7.0% (119/1700) of cases were over‐graded and 22.5% (383/1700) were undergraded compared to the consensus diagnoses.^64^

Studies by Harnden et al.,^65^ Griffiths et al.,^66^ De la Taille et al.,^67^ and Lessels et al.^68^ examined reproducibility across varying GS categories. Harnden and Griffiths both used diagnostic categories of GS 2−4, 5−6, 7, and 8−10. Griffiths found poor agreement among 24 pathologists for GS 5‐6 lesions (overall *ϰ*: 0.27).^66^ In Harnden's study, for the 5 consensus GS5‐6 cases, 40 pathologists (8 uropathologists, 32 other pathologists) agreed with the consensus diagnosis in 79.5% (136/171) of classifications.^65^ In De la Taille's study with diagnostic categories of GS 4−6, 7, and 8−10, there was only modest agreement among 10 pathologists who evaluated 104 cases (*ϰ* ranged from 0.28 to 0.54), with the number of cases classified as GS 4−6 ranging from 18.5% to 68.4% among participants.^67^ In Lessells et al's study of 46 cases, there was modest agreement among 12 pathologists for classifications across three diagnostic categories (GS 2−4, 5−6, and 7−10; overall *ϰ*: 0.41).^68^

Finally the study by McKenney examined recognition of GP 3−5.^69^ Among 11 genitourinary pathologists, there was moderate agreement for 17 cases with classic patterns (overall *ϰ*: 0.76, 95% CI: 0.59−0.90). However, there was poor agreement for a second set of 17 cases with “patterns with a potential reproducibility problem” such as tangentially sectioned GP3 versus GP4 with poorly formed glands (overall *ϰ*: 0.27, 95% CI: 0.15−0.42).

### 3.4 Is there evidence of diagnostic drift over time in the histopathological diagnostic threshold of GS 6 prostate lesions? (*n* = 4, Table 4, Figure 2)

**Figure 2:**
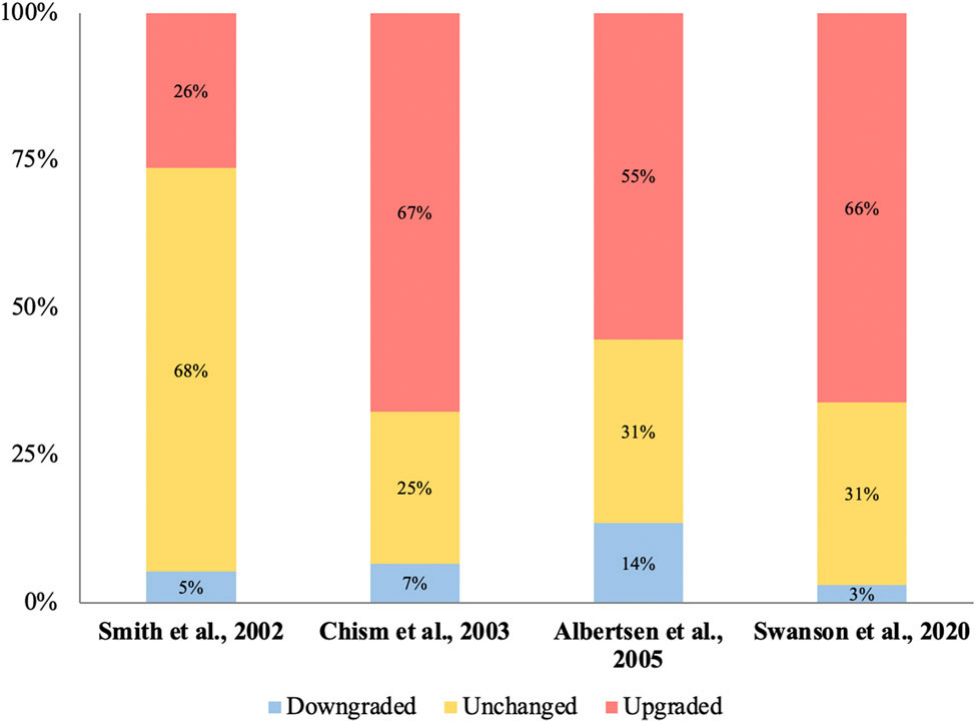
Diagnostic drift: changes in Gleason scoring when pathology slides are reassessed years later. Smith^72^: initial diagnosis 1989−1991, reassessment 2000. Chism^73^: initial diagnosis 1987−1993, reassessment 1998. Albertsen^71^: initial diagnosis 1990−1992, reassessment 2002−2004. Swanson^74^: initial diagnosis 1985−1995, reassessment period not reported. [Color figure can be viewed at wileyonlinelibrary.com]

We screened the titles and abstracts of 233 articles identified through our database searches, resulting in 28 full texts to screen, of which 24 were excluded (flow diagram in Supporting Information: 2d, reasons for exclusion in Supporting Information: 3d). We included 4 studies in our narrative synthesis (Table 4 and Figure 2). Two studies were rated as low risk of bias and 2 studies were rated as high risk of bias (Supporting Information: 4d).

**Table 4:** Diagnostic drift studies.^a^

Author	Year	Country	Period 1		Period 2		Total number of cases	Cases with an original diagnosis of Gleason score 6 or ≤6	Diagnostic thresholds for reclassification	Number of Gleason score 6 cases or total number of cases reclassified (%)		Overall risk of bias^b^
			Diagnostic period	Pathologists (*n*)	Diagnostic period	Pathologists (*n*)				Upgraded	Downgraded	
Albertsen^71^	2005	USA	1990−1992	Type and number not specified	2002−2004	Pathologist with prostate expertise (1)	1858 biopsies	454 GS6	Gleason scores 2−4, 5, 6, 7, 8, 9, and 10	171/454 GS6 (38%)	22/454 GS6 (5%)	Low
Smith^72^	2002	USA	1989−1991	Genito‐urinary pathologists (2)	2000	Genito‐urinary pathologists (2)^c^	23 biopsies, 15 RP specimens	16 biopsies, 9 RP specimens GS ≤6	Gleason scores ≤6, 7, and ≥8	8/23 (35%) biopsies, 2/15 (13%) RP specimens	2/23 (9%) biopsies, 0/15 (0%) RP specimens	Low
Chism^73^	2003	USA	1987−1993	Urologic pathologist, number not specified	1998	Pathologist (1)	106 biopsies	23 GS6	Gleason scores 2−5, 6, 7, and 8−10	17/23 GS6 (74%)	2/23 GS6 (9%)	High
Swanson^74^	2020	USA	1985−1995	Type and number not specified	Does not report	Genito‐urinary pathologist (1)	499 RP specimens	180 GS ≤6	Gleason scores ≤6, 7, and ≥8	329/499 (66%)	15/499 (3%)	High

Abbreviation: RP, radical prostatectomy.

^a^ Diagnostic drift studies refer to studies with two or more independent readings of the same histopathological slides by pathologists at two or more time points for the purposes of diagnostic classification.

^b^ See Supporting Information: Figure S4d for complete risk of bias assessment.

^c^ Same as original readers.

All studies provided direct evidence of the upgrading of GS6 prostate lesions over time. Chism et al.^73^ reported the diagnoses of 106 prostate biopsies made by a single pathologist in 1998 blinded to the original diagnoses given by urologic pathologists in 1987−1993. Of the 23 cases with an original diagnosis of GS6, 74% (17/23) were upgraded and 9% (2/23) were downgraded. Smith et al.^72^ reported the diagnoses of two genitourinary pathologists in 2000 blinded to their own original diagnoses of prostate biopsies and radical prostatectomy specimens in 1989−1991. Of the specimens originally diagnosed in 1989−1991, 35% (8/23) of biopsies were upgraded and 9% (2/23) were downgraded, and 13% (2/15) radical prostatectomy specimens were upgraded and 0% (0/15) were downgraded. Albertsen et al.^71^ reported the diagnoses made by one pathologist in 2002−2004 of 1858 prostate biopsies, blinded to the original diagnoses made in 1990−1992. Of the 454 cases with an original diagnosis of GS6, 38% (171/454) were upgraded and 5% (22/454) were downgraded. Finally, Swanson published a report in 2020^74^ of the diagnoses for 499 radical prostatectomy specimens made by a single genitourinary pathologist blinded to the original diagnoses made in 1985−1995. Overall, 66% (329/499) of specimens were upgraded and 3% (15/499) were downgraded.

## 4 DISCUSSION

This review found robust evidence to inform discussion of possible recalibration of diagnostic thresholds, or use of alternative labels, for low‐risk prostate cancer. RCTs and cohort studies demonstrate that active surveillance is a safe option for very low‐risk and low‐risk prostate cancers, with extremely low rates of metastasis and prostate cancer‐specific mortality over follow‐up periods up to 15 years. However after 5 years, up to 9% of patients terminate active surveillance without medical indication to do so. The indolent nature of these lesions is further supported by the high rates of subclinical cancer in men who died from other causes. Reproducibility of low‐risk prostate cancer diagnosis was high among pathologists and specialist uropathologists in the most recent study (published in 2020) rated at low risk of bias. It was more variable in other studies rated at moderate risk of bias. Finally, there is clear evidence of diagnostic drift over time, whereby a higher GS would be applied to the same prostate lesion when rereviewed years after the original diagnostic classification. In the most recent study published in 2020, 66% of cases were upgraded and only 3% downgraded compared to the original classification in 1985−1995, indicating that risk of upgrading may be increasing with time. This trend is likely due to both explicit changes to diagnostic thresholds and implicit changes in thresholds used by pathologists. Explicit changes to diagnostic thresholds include the abolition of Gleason grades 1 and 2, and the expanded inclusion criteria for GP4 at the 2005 ISUP consensus conference with further GP4 definition expansion at the 2014 ISUP consensus conference. These changes likely represent significant contributors to the upgrading of pre‐2005 cases that were reevaluated post‐2005.^75^ Diagnostic scrutiny may also result in grade migration over time. Increased prostate sampling associated with the transition to transperineal biopsies or with repeat sampling in active surveillance protocols increase the chance of obtaining a biopsy core containing GP4.

The findings of this review are consistent with, and build upon, published evidence reporting favorable outcomes from active surveillance for low‐risk prostate cancer, and the large burden of subclinical disease found in autopsy studies. It is the first review to also collate evidence on the reproducibility and shifting diagnostic thresholds for such lesions. The strengths of this review lie in its methodological rigor, including a comprehensive search of the literature supplemented by articles suggested by content experts, and two reviewers completing all steps, including risk of bias assessment. The cross‐disciplinary authorship group, including uropathologists and a treating clinician, helps to ensure our findings are relevant to those practicing in the field. The limitations of this review include the high risk of bias and inadequate reporting in some studies.

There have been a number of calls to adopt non‐“cancer” labels for low‐risk prostate cancer to reduce potential harms from overdiagnosis and overtreatment.^28^, ^29^, ^76^, ^78^, ^79^ However, some pathologists argue against such a change on biological grounds, saying that GG1 lesions are morphologically indistinguishable from GG2‐5 lesions, share many molecular hallmarks of prostate cancer, and therefore these lesions should retain the “cancer” label.^80^, ^81^ They also cite a high prevalence (up to 30%) of sampling variation (e.g., where a GG1 lesion is detected on biopsy in an individual harboring a higher‐risk cancer).^80^, ^82^ Others have countered by arguing that even if GG1 lesions appear morphologically similar to higher‐grade lesions and/or invade the stroma, they consistently behave as precancerous lesions—with extraprostatic extension and metastasis being extremely rare.^29^ The low rates of metastasis and prostate cancer deaths in men undergoing active surveillance in this review support this characterization of a more benign clinical behavior. Further, among patients who are candidates for active surveillance based on clinical features, pathological upgrading (i.e., discovery of higher‐grade lesions after the initial biopsy) has been found to have limited long‐term effects on clinical outcomes.^29^, ^83^ New methods of detection (e.g., combined MRI‐targeted and systematic biopsies) may also help to minimize the incidence of sampling variation.^84^

Another key component of this debate is the hypothesized impact of relabeling lesions as noncancerous on patients' attitudes and behaviors. A common fear raised by those against such a move is that without the “cancer” label, patients may be less engaged with follow‐up screening practices, possibly leading to poorer clinical outcomes. They point to suboptimal adherence to active surveillance protocols in both community and clinical trial settings, with biopsy adherence rates decreasing over time on surveillance.^85^, ^86^, ^87^ A change in label is also argued to be unnecessary, as the shift in terminology from GS6 to GG1 communicates the low‐risk nature of the lesion, and uptake of active surveillance has been increasing.^80^ Countering these concerns, others hypothesize that relabeling cancerous lesions may decrease patient and family anxiety, as well as other psychosocial impacts on relationships, employment, and insurance policies. This may further facilitate uptake of active surveillance, as well as preventing termination of active surveillance without medical indication.^78^

## 5 CONCLUSION

The decision whether to relabel low‐risk prostate cancer as a noncancerous lesion (by adopting a new label or recalibration of the diagnostic threshold) is a complex issue. Many aspects of this discussion are evolving, such as the integration of multiparametric MRI in diagnostic protocols,^88^ and the use of artificial intelligence to increase reproducibility of histopathological diagnoses. Further robust debate is needed in both clinical and pathology communities, informed by evidence such as that collected here.

## AUTHOR CONTRIBUTIONS

*Conceptualization*: Brooke Nickel and Katy J. L. Bell. *Methodology*: Brooke Nickel and Katy J. L. Bell. *Investigation*: Caitlin R. Semsarian and Tara Ma. *Data Curation*: Caitlin R. Semsariana and Tara Ma. *Writing—Original Draft*: Caitlin R. Semsarian and Katy J. L. Bell. *Writing—Review and Editing*: All authors. *Visualization*: Caitlin R. Semsariana and Katy J. L. Bell. *Supervision*: Katy J. L. Bell. *Project Administration*: Caitlin R. Semsarian and Katy J. L. Bell. *Funding Acquisition*: Katy J. L. Bell.

## CONFLICT OF INTEREST STATEMENT

The authors declare no conflict of interest.

## Supporting information

Supplementary information.

## Data Availability

The data that support the findings of this study are openly available and can be retrieved from databases PubMed and EMBASE.
