# Supplementary material for: Low‐risk prostate lesions: An evidence review to inform discussion on losing the “cancer” label
Source: Prostate. 2023 Feb 22;83(6):498–515. doi: 10.1002/pros.24493 (PMC10952636; doi:10.1002/pros.24493)
Supplement: Supplementary file 1 — Supplementary information. [file PROS-83-498-s001.docx]

**SUPPLEMENT 1: Search strategies.**

1. **Active surveillance**

**EMBASE**

‘watchful waiting’/exp AND ‘active surveillance’.mp AND (prostate.mp OR prostatic.mp), limit to *human* and *article*

**PubMed**

“Watchful Waiting”[Mesh] AND ("Follow-Up Studies"[Mesh] OR "Predictive Value of Tests"[Mesh] OR "Prospective Studies"[Mesh]) AND ("active surveillance"[Text Word] OR "active monitoring"[Text Word]) AND (prostate[Text Word] OR prostatic[Text Word]), limit to *humans* and *journal article*

1. **Reproducibility**

**EMBASE**

(reproducibility.mp OR interobserver.mp OR inter-observer.mp OR (discordance.mp AND diagnosis.mp) OR (observer.mp AND accuracy.mp) OR (case.mp AND interpretation.mp) OR (comparison.mp AND review.mp) AND (histopatholog*.mp OR histomorpholog*.mp OR (histolog*.mp AND (classification.mp OR grading.mp)) OR ‘Gleason grading’.mp) AND pathologist*.mp AND (prostate.mp OR prostatic.mp OR Gleason.mp), limit to *human* and *article*

**PubMed**

(reproducibility[Text Word] OR interobserver[Text Word] OR inter-observer[Text Word] OR (discordance[Text Word] AND diagnosis[Text Word]) OR (observer[Text Word] AND accuracy[Text Word]) OR (case[Text Word] AND interpretation[Text Word]) OR (comparison[Text Word] AND review[Text Word])) AND (histopatholog*[Text Word] OR histomorpholog*[Text Word] OR ((histolog*[Text Word] OR pathologist*[Text Word]) AND (classification[Text Word] OR grading[Text Word)) OR "Gleason grading"[Text Word]) AND pathologist*[Text Word] AND (prostate[Text Word] OR prostatic[Text Word] OR Gleason[Text Word]), limit to *humans* and *journal article*

1. **Autopsy studies**

**EMBASE**

((autopsy.mp OR autopsies.mp) AND (population.mp OR study.mp OR series.mp)) AND (incidental.mp OR incidence.mp OR prevalence.mp OR latent.mp OR frequency.mp) AND (prostate.mp OR prostatic.mp), limit to *human* and *article*

**PubMed**

((autopsy[Text Word] OR autopsies[Text Word]) AND (population[Text Word] OR study[Text Word] OR series[Text Word])) AND (incidental[Text Word] OR incidence[Text Word] OR prevalence[Text Word] OR latent[Text Word] OR frequency[Text Word]) AND (prostate[Text Word] OR prostatic[Text Word]), limit to *humans* and *journal article*

1. **Diagnostic drift**

**EMBASE**

(‘diagnostic drift’.mp OR ‘stage migration’.mp OR ‘grade inflation’.mp OR (‘Will Rogers’.mp AND (effect.mp OR phenomenon.mp))) AND (prostate.mp OR Gleason.mp), limit to *human* and *article*

**PubMed**

("diagnostic drift"[Text Word] OR "stage migration"[Text Word] OR "grade inflation"[Text Word] OR ("Will Rogers"[Text Word] AND (effect[Text Word] OR phenomenon[Text Word]))) AND (prostate[Text Word] OR Gleason[Text Word]), limit to *humans* and *journal article*

**SUPPLEMENT 2: Literature search and study selection process.**

(a) Active surveillance search. (b) Autopsy study search. (c) Reproducibility study search. (d) Diagnostic drift search.

(a)


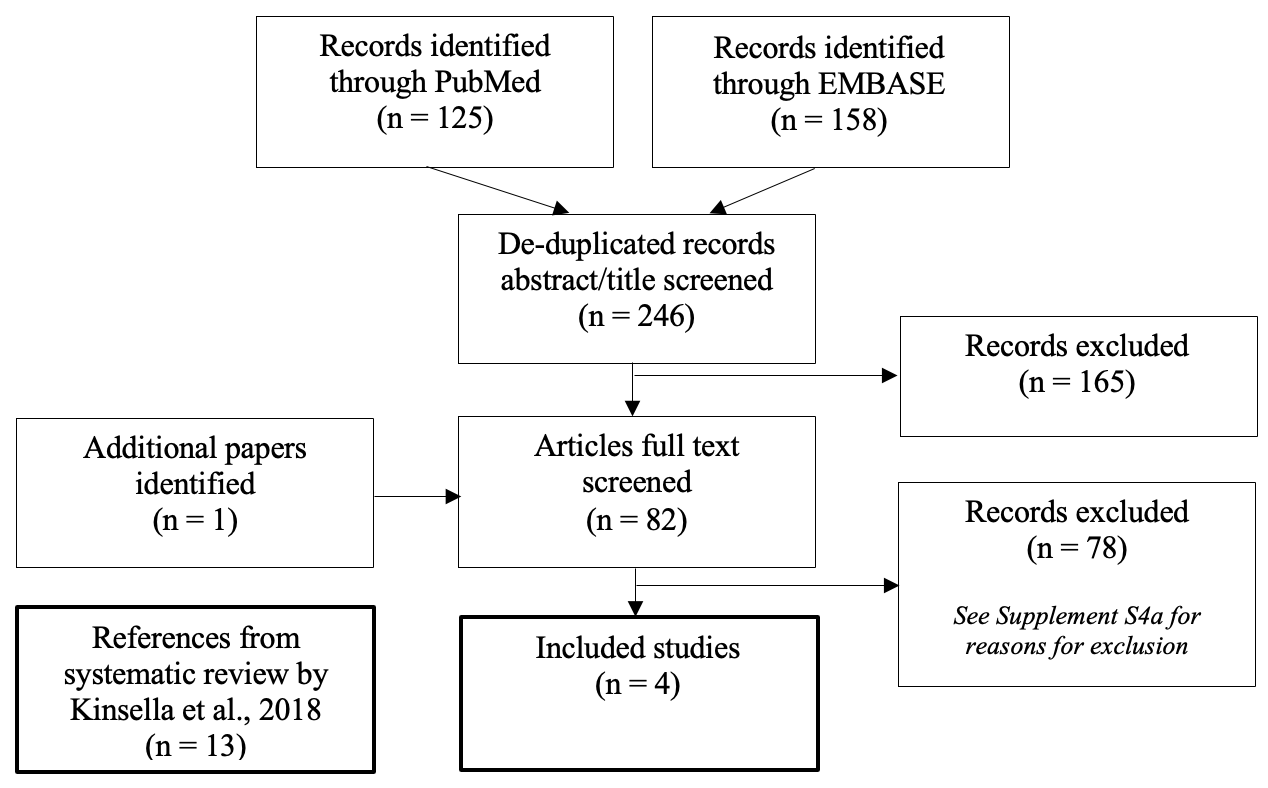


(b)


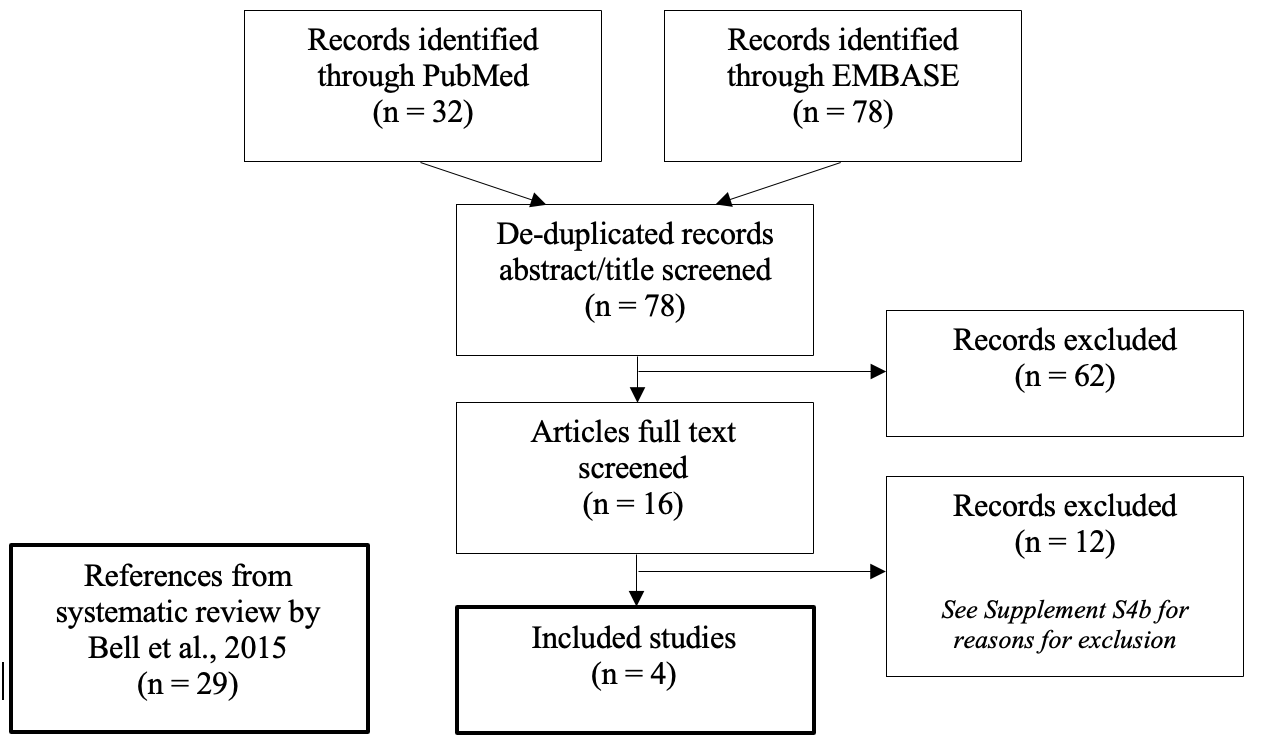


(c)


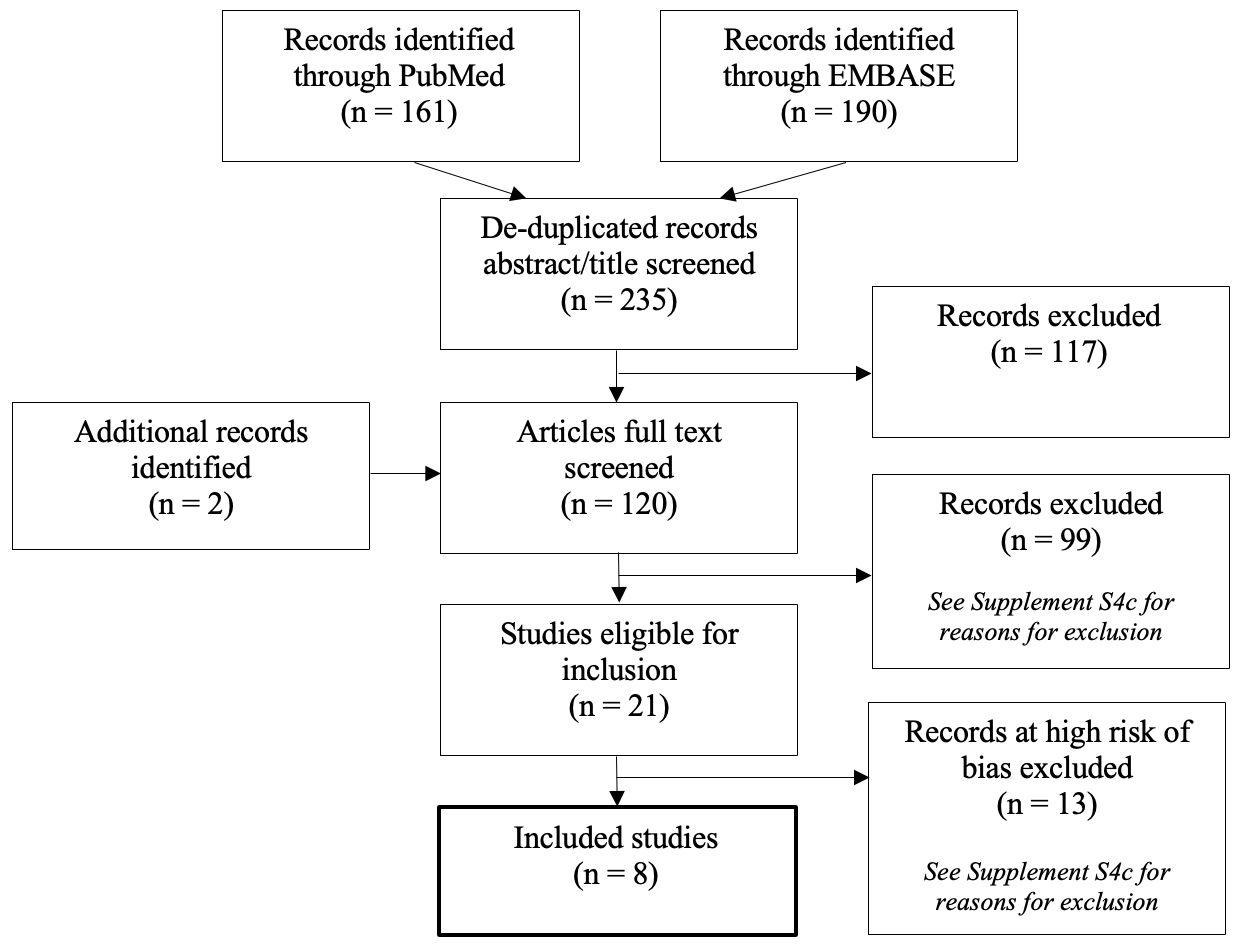


(d)

**
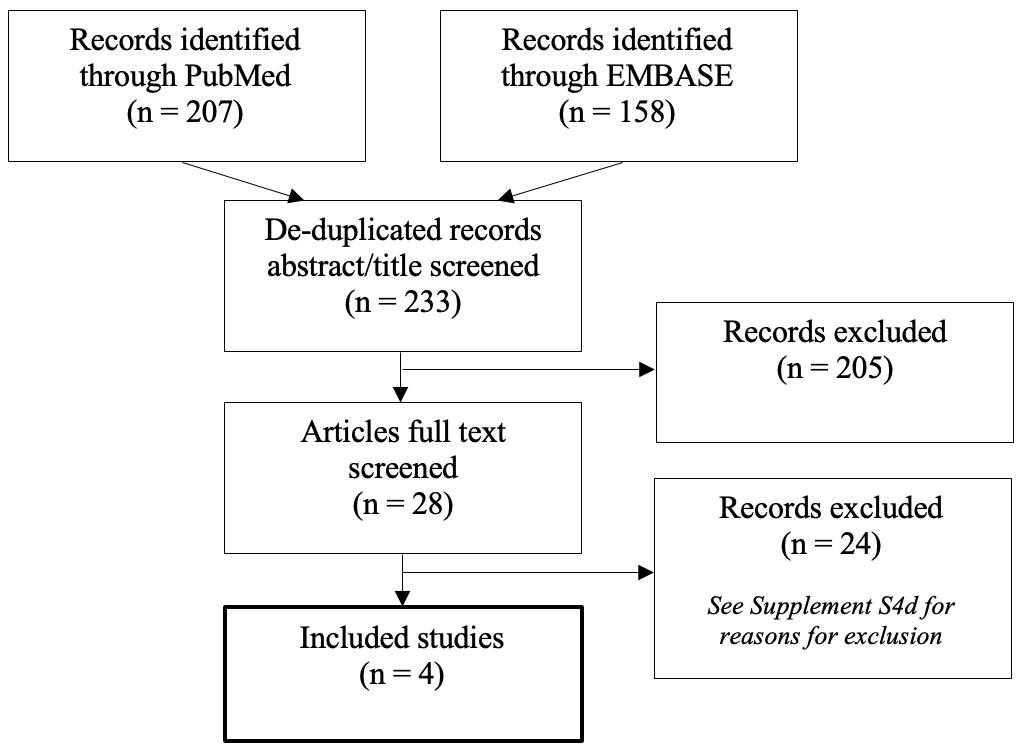
**

**SUPPLEMENT 3: Reasons for exclusion of articles at full text screening.**

**Table S3a: Reasons for records excluded from active surveillance studies (n=78).**

| No. | Article | Reasons for exclusion |
| --- | --- | --- |
| 1 | Abdel-Rahman, O. 2019. 'Outcomes of clinically localized prostate cancer patients managed with initial monitoring approach versus upfront local treatment: a North American population-based study', *Clinical and Translational Oncology*, 21(12): 1673-79. | Retrospective study |
| 2 | Ahmad, A. E., P. O. Richard, R. Leao, M. Hajiha, L. J. Martin, M. Komisarenko, R. Grewal, H. Goldberg, S. Salem, K. Jain, A. Oliaei, I. Horyn, N. Timilshina, A. Zlotta, R. Hamilton, G. Kulkarni, N. Fleshner, S. M. H. Alibhaic, and A. Finelli. 2020. 'Does Time Spent on Active Surveillance Adversely Affect the Pathological and Oncologic Outcomes in Patients Undergoing Delayed Radical Prostatectomy?', *The Journal of urology*, 204(3): 476-82. | Retrospective study |
| 3 | Albers, P., T. Wiegel, H. Schmidberger, R. Bussar-Maatz, M. Härter, G. Kristiansen, P. Martus, C. Meisner, S. Wellek, K. Grozinger, P. Renner, M. Burmester, F. Schneider, and M. Stöckle. 2021. 'Termination rates and histological reclassification of active surveillance patients with low- and early intermediate-risk prostate cancer: results of the PREFERE trial', *World J Urol*, 39: 65-72. | Follow-up <5 years |
| 4 | Balakrishnan, A. S., J. E. Cowan, M. R. Cooperberg, K. Shinohara, H. G. Nguyen, and P. R. Carroll. 2019. 'Evaluating the Safety of Active Surveillance: Outcomes of Deferred Radical Prostatectomy after an Initial Period of Surveillance', *J Urol*, 202: 506-10. | Analysis of patients who converted to radical prostatectomy |
| 5 | Besasie, B. D., A. G. Sunnapwar, F. Gao, D. Troyer, G. D. Clarke, H. White, P. T. Fox, A. Dale, A. Wheeler, and M. A. Liss. 2021. 'Restriction Spectrum Imaging-Magnetic Resonance Imaging to Improve Prostate Cancer Imaging in Men on Active Surveillance', *J Urol*, 206: 44-51. | Follow-up <5 years |
| 6 | Borghesi, M., L. Bianchi, U. Barbaresi, V. Vagnoni, B. Corcioni, C. Gaudiano, M. Fiorentino, F. Giunchi, F. Chessa, M. Garofalo, A. Bertaccini, S. Angelini, A. Ercolino, C. Casablanca, M. Droghetti, R. Golfieri, and R. Schiavina. 2021. 'Diagnostic performance of MRI/TRUS fusion-guided biopsies vs. systematic prostate biopsies in biopsy-naïve, previous negative biopsy patients and men undergoing active surveillance', *Minerva Urol Nephrol*, 73: 357-66. | Not an active surveillance study |
| 7 | Botejue, M., D. Abbott, J. Danella, C. Fonshell, S. Ginzburg, T. J. Guzzo, T. Lanchoney, B. Marlowe, J. D. Raman, M. Smaldone, J. J. Tomaszewski, E. J. Trabulsi, R. G. Uzzo, and A. C. Reese. 2019. 'Active Surveillance as Initial Management of Newly Diagnosed Prostate Cancer: Data from the PURC', *Journal of Urology*, 201(5): 929-35. | Retrospective study |
| 8 | Bratt, O., E. Holmberg, O. Andrén, S. Carlsson, L. Drevin, E. Johansson, A. Josefsson, M. Nyberg, J. Sandberg, P. Stattin, and D. Robinsson. 2019. 'The Value of an Extensive Transrectal Repeat Biopsy with Anterior Sampling in Men on Active Surveillance for Low-risk Prostate Cancer: A Comparison from the Randomised Study of Active Monitoring in Sweden (SAMS)', *Eur Urol*, 76: 461-66. | Follow-up <5 years |
| 9 | Bruinsma, S. M., D. Nieboer, M. J. Roobol, C. H. Bangma, J. F. M. Verbeek, V. Gnanapragasam, M. Van Hemelrijck, M. Frydenberg, L. S. Lee, R. Valdagni, C. Logothetis, and E. W. Steyerberg. 2021. 'Risk-Based Selection for Active Surveillance: Results of the Movember Foundation's Global Action Plan Prostate Cancer Active Surveillance (GAP3) Initiative', *The Journal of urology*, 206(1): 62-68. | Retrospective study |
| 10 | Caglic, I., N. Sushentsev, V. J. Gnanapragasam, E. Sala, N. Shaida, B. C. Koo, V. Kozlov, A. Y. Warren, C. Kastner, and T. Barrett. 2021. 'MRI-derived PRECISE scores for predicting pathologically-confirmed radiological progression in prostate cancer patients on active surveillance', *European Radiology*, 31(5): 2696-705. | Retrospective study |
| 11 | Carlsson, S., N. Benfante, R. Alvim, D. D. Sjoberg, A. Vickers, V. E. Reuter, S. W. Fine, H. A. Vargas, M. Wiseman, M. Mamoor, B. Ehdaie, V. Laudone, P. Scardino, J. Eastham, and K. Touijer. 2020. 'Long-Term Outcomes of Active Surveillance for Prostate Cancer: The Memorial Sloan Kettering Cancer Center Experience', *J Urol*, 203: 1122-27. | Retrospective study |
| 12 | Carlsson, S., N. Benfante, R. Alvim, D. D. Sjoberg, A. Vickers, V. E. Reuter, S. W. Fine, H. A. Vargas, M. Wiseman, M. Mamoor, B. Ehdaie, V. Laudone, P. Scardino, J. Eastham, and K. Touijer. 2020. 'Risk of Metastasis in Men with Grade Group 2 Prostate Cancer Managed with Active Surveillance at a Tertiary Cancer Center', *J Urol*, 203: 1117-21. | Gleason score 7 lesions only |
| 13 | Carlsson, S., O. Bratt, D. Kristiansson, and F. Jäderling. 2020. 'The value of a first MRI and targeted biopsies after several years of active surveillance for low-risk prostate cancer - results from the SAMS trial', *Scand J Urol*, 54: 318-22. | <100 patients |
| 14 | Chu, C. E., P. E. Lonergan, S. L. Washington, J. E. Cowan, K. Shinohara, A. C. Westphalen, P. R. Carroll, and M. R. Cooperberg. 2020. 'Multiparametric Magnetic Resonance Imaging Alone is Insufficient to Detect Grade Reclassification in Active Surveillance for Prostate Cancer', *Eur Urol*, 78: 515-17. | Follow-up <5 years |
| 15 | Chu, C. E., J. E. Cowan, V. Fasulo, S. L. Washington, 3rd, C. de la Calle, J. Shoemaker, and P. R. Carroll. 2021. 'The Clinical Significance of Multiple Negative Surveillance Prostate Biopsies for Men on Active Surveillance-Does Cancer Vanish or Simply Hide?', *J Urol*, 205: 109-14. | Retrospective study |
| 16 | Cooley, L. F., A. A. Emeka, T. J. Meyers, P. R. Cooper, D. W. Lin, A. Finelli, J. A. Eastham, C. J. Logothetis, L. S. Marks, D. Vesprini, S. L. Goldenberg, C. S. Higano, C. P. Pavlovich, J. M. Chan, T. M. Morgan, E. A. Klein, D. A. Barocas, S. Loeb, B. T. Helfand, D. M. Scholtens, J. S. Witte, and W. J. Catalona. 2021. 'Factors Associated with Time to Conversion from Active Surveillance to Treatment for Prostate Cancer in a Multi-Institutional Cohort', *J Urol*, 206: 1147-56. | Retrospective study |
| 17 | Dai, C., V. Ganesan, Y. A. Nyame, N. Almassi, D. J. Greene, D. Hettel, C. Magi-Galluzzi, M. Gong, J. S. Jones, A. J. Stephenson, R. K. Berglund, and E. A. Klein. 2019. 'Older Age at Diagnosis and Initial Disease Volume Predict Grade Reclassification Risk on Confirmatory Biopsy in Patients Considered for Active Surveillance', *Urology*, 130: 106-12. | Retrospective study |
| 18 | Detsky, J. S., A. F. Ghiam, A. Mamedov, K. Commisso, A. Commisso, L. Zhang, S. Liu, L. Klotz, A. Loblaw, and D. Vesprini. 2020. 'Impact of Biopsy Compliance on Outcomes for Patients on Active Surveillance for Prostate Cancer', *J Urol*, 204: 934-40. | Retrospective study |
| 19 | Druskin, S. C., M. Mamawala, J. J. Tosoian, J. I. Epstein, C. P. Pavlovich, H. B. Carter, and B. J. Trock. 2019. 'Older age predicts biopsy and radical prostatectomy grade reclassification to aggressive prostate cancer in men on active surveillance', *Journal of Urology*, 201(1): 98-104. | Follow-up <5 years |
| 20 | Evans, M. A., J. L. Millar, A. Earnest, M. Frydenberg, I. D. Davis, D. G. Murphy, P. A. Kearns, and S. M. Evans. 2018. 'Active surveillance of men with low risk prostate cancer: Evidence from the prostate cancer outcomes registrye - Victoria', *Medical Journal of Australia*, 208(10): 439-43. | Clinical outcomes of relevance not reported |
| 21 | Fernández-Conejo, G., E. de la Peña, V. Hernández, E. Pérez-Fernández, and C. Llorente. 2019. 'Transition from active surveillance to observation in prostate cancer patients older than 75 years. A long follow-up series', *Actas Urol Esp (Engl Ed)*, 43: 378-83. | Retrospective study |
| 22 | Fiorella, D., J. L. Marenco, J. M. Mascarós, Á Borque-Fernando, L. M. Esteban, A. Calatrava, B. Pastor, J. A. López-Guerrero, and J. Rubio-Briones. 2021. 'Role of PCA3 and SelectMDx in the optimization of active surveillance in prostate cancer', *Actas Urol Esp (Engl Ed)*, 45: 439-46. | <100 patients |
| 23 | Frandon, J., E. Bey, A. Hamard, H. Mohammad, S. Gonzalez, J. Greffier, T. Chevallier, H. de Forges, J. P. Beregi, and S. Droupy. 2021. 'Early Results of Unilateral Prostatic Artery Embolization as a Focal Therapy in Patients with Prostate Cancer under Active Surveillance: Cancer Prostate Embolisation, a Pilot Study', *J Vasc Interv Radiol*, 32: 247-55. | <100 patients |
| 24 | Gielchinsky, I., J. Chang, T. Cusick, W. Delprado, Q. Nguyen, C. Yuen, R. Savdie, M. Böhm, A. M. Haynes, M. J. Scheltema, and P. D. Stricker. 2018. 'Prostate cancer in 432 men aged <50 years in the prostate-specific antigen era: a new outlook', *BJU Int*, 122 Suppl 5: 35-41. | <100 patients |
| 25 | Gill, I. S., A. R. Azzouzi, M. Emberton, J. A. Coleman, E. Coeytaux, A. Scherz, and P. T. Scardino. 2018. 'Randomized Trial of Partial Gland Ablation with Vascular Targeted Phototherapy versus Active Surveillance for Low Risk Prostate Cancer: Extended Followup and Analyses of Effectiveness', *J Urol*, 200: 786-93. | Follow-up <5 years |
| 26 | Gregg, J. R., D. S. Surasi, A. Childs, N. Moll, J. F. Ward, J. Kim, C. R. Daniel, C. Logothetis, T. Bathala, and J. W. Davis. 2021. 'The Association of Periprostatic Fat and Grade Group Progression in Men with Localized Prostate Cancer on Active Surveillance', *J Urol*, 205: 122-28. | Retrospective study |
| 27 | Ha, J. Y., T. J. Shin, W. Jung, B. H. Kim, C. H. Park, and C. I. Kim. 2017. 'Updated clinical results of active surveillance of very-low-risk prostate cancer in Korean men: 8 years of follow-up', *Investigative and Clinical Urology*, 58(3): 164-70. | <100 patients |
| 28 | Halstuch, D., Y. Ber, D. Kedar, S. Golan, J. Baniel, and D. Margel. 2020. 'Short-Term Outcomes of Active Surveillance for Low Risk Prostate Cancer among Men with Germline DNA Repair Gene Mutations', *J Urol*, 204: 707-13. | <100 patients |
| 29 | Hamdy, F. C., J. L. Donovan, J. A. Lane, M. Mason, C. Metcalfe, P. Holding, J. Wade, S. Noble, K. Garfield, G. Young, M. Davis, T. J. Peters, E. L. Turner, R. M. Martin, J. Oxley, M. Robinson, J. Staffurth, E. Walsh, J. Blazeby, R. Bryant, P. Bollina, J. Catto, A. Doble, A. Doherty, D. Gillatt, V. Gnanapragasam, O. Hughes, R. Kockelbergh, H. Kynaston, A. Paul, E. Paez, P. Powell, S. Prescott, D. Rosario, E. Rowe, and D. Neal. 2020. 'Active monitoring, radical prostatectomy and radical radiotherapy in PSA-detected clinically localised prostate cancer: the ProtecT three-arm RCT', *Health Technol Assess*, 24: 1-176. | ProtecT cohort 10-year outcomes already published in NEJM and captured in Kinsella systematic review, no new outcome data presented here |
| 30 | Hamoen, E. H. J., C. M. A. Hoeks, D. M. Somford, I. M. van Oort, H. Vergunst, J. R. Oddens, G. A. Smits, L. P. Bokhorst, J. A. Witjes, M. M. Rovers, C. A. Hulsbergen-van de Kaa, and J. O. Barentsz. 2019. 'Value of Serial Multiparametric Magnetic Resonance Imaging and Magnetic Resonance Imaging-guided Biopsies in Men with Low-risk Prostate Cancer on Active Surveillance After 1 Yr Follow-up', *Eur Urol Focus*, 5: 407-15. | Follow-up <5 years |
| 31 | Herden, J., E. A. Boedefeld, and L. Weissbach. 2020. 'Noninvasive treatment of organ-confined prostate cancer in elderly patients-results of the HAROW study. [German]', *Urologe*, 59(4): 450-60. | Sub-group analysis of HAROW cohort |
| 32 | Herden, J., A. Schwarte, E. A. Boedefeld, and L. Weissbach. 2021. 'Active Surveillance for Incidental (cT1a/b) Prostate Cancer: Long-Term Outcomes of the Prospective Noninterventional HAROW Study', *Urol Int*, 105: 428-35. | <100 patients |
| 33 | Hoffman, K. E., D. F. Penson, Z. Zhao, L. C. Huang, R. Conwill, A. A. Laviana, D. D. Joyce, A. N. Luckenbaugh, M. Goodman, A. S. Hamilton, X. C. Wu, L. E. Paddock, A. Stroup, M. R. Cooperberg, M. Hashibe, B. B. O'Neil, S. H. Kaplan, S. Greenfield, T. Koyama, and D. A. Barocas. 2020. 'Patient-Reported Outcomes Through 5 Years for Active Surveillance, Surgery, Brachytherapy, or External Beam Radiation With or Without Androgen Deprivation Therapy for Localized Prostate Cancer', *Jama*, 323: 149-63. | Clinical outcomes of relevance not reported |
| 34 | Hongo, F., K. Okihara, K. Kitamura, A. Fujihara, Y. Yamada, T. Shiraishi, E. Konishi, and O. Ukimura. 2019. 'Prostate cancer meeting the Japanese active surveillance criteria and diagnosed by community-based prostate-specific antigen screening: A 21-year follow-up study', *Int J Urol*, 26: 827-32. | Retrospective study |
| 35 | Houédé, N., X. Rébillard, S. Bouvet, S. Kabani, P. Fabbro-Peray, B. Trétarre, and F. Ménégaux. 2020. 'Impact on quality of life 3 years after diagnosis of prostate cancer patients below 75 at diagnosis: an observational case-control study', *BMC Cancer*, 20: 757. | Not an active surveillance study |
| 36 | Kariburyo, F., Y. Wang, I. E. Cheng, L. Wang, D. Morgenstern, L. Xie, E. Meadows, J. Danella, and M. L. Cher. 2018. 'Observation versus treatment among men with favorable risk prostate cancer in a community-based integrated health care system: a retrospective cohort study', *BMC Urol*, 18: 55. | Retrospective study |
| 37 | Klotz, L., G. Pond, A. Loblaw, L. Sugar, M. Moussa, D. Berman, T. Van der Kwast, D. Vesprini, L. Milot, M. Kebabdjian, N. Fleshner, S. Ghai, J. Chin, and M. Haider. 2020. 'Randomized Study of Systematic Biopsy Versus Magnetic Resonance Imaging and Targeted and Systematic Biopsy in Men on Active Surveillance (ASIST): 2-year Postbiopsy Follow-up', *Eur Urol*, 77: 311-17. | Follow-up <5 years |
| 38 | Kum, F., K. Beckmann, H. Aya, S. Singh, P. Sandhu, S. Sra, J. Rusere, G. Zisengwe, A. Santaolalla, P. Cathcart, B. Challacombe, C. Brown, R. Popert, P. Dasgupta, M. Van Hemelrijck, and O. Elhage. 2021. 'Presentation, follow-up, and outcomes among African/Afro-Caribbean men on active surveillance for prostate cancer: experiences of a high-volume UK centre', *Prostate Cancer and Prostatic Diseases*, 24(2): 549-57. | Retrospective study |
| 39 | Laviana, A. A., Z. Zhao, L. C. Huang, T. Koyama, R. Conwill, K. Hoffman, M. Goodman, A. S. Hamilton, X. C. Wu, L. E. Paddock, A. Stroup, M. R. Cooperberg, M. Hashibe, B. B. O'Neil, S. H. Kaplan, S. Greenfield, D. F. Penson, and D. A. Barocas. 2020. 'Development and Internal Validation of a Web-based Tool to Predict Sexual, Urinary, and Bowel Function Longitudinally After Radiation Therapy, Surgery, or Observation', *Eur Urol*, 78: 248-55. | Not an active surveillance study, clinical outcomes of relevance not reported |
| 40 | Le Paih, J. P., F. Bladou, C. Klein, B. Rouget, M. Hugo, J. M. Ferriere, H. Bensadoun, J. C. Bernhard, G. Capon, and G. Robert. 2020. 'Predictive factors of active surveillance interruption for prostate cancer after 5years of follow-up. [French]', *Progres en urologie : journal de l'Association francaise d'urologie et de la Societe francaise d'urologie*, 30(8-9): 463-71. | Article in French, follow-up <5 years |
| 41 | Liss, M. A., L. F. Newcomb, Y. Zheng, M. P. Garcia, C. P. Filson, H. Boyer, J. D. Brooks, P. R. Carroll, M. R. Cooperberg, W. J. Ellis, M. E. Gleave, F. M. Martin, T. Morgan, P. S. Nelson, A. A. Wagner, I. M. Thompson, Jr., and D. W. Lin. 2020. 'Magnetic Resonance Imaging for the Detection of High Grade Cancer in the Canary Prostate Active Surveillance Study', *J Urol*, 204: 701-06. | Study of MRI in the Canary PASS cohort |
| 42 | Lonergan, P. E., S. L. Washington, 3rd, J. E. Cowan, S. Zhao, H. G. Nguyen, K. Shinohara, M. R. Cooperberg, and P. R. Carroll. 2020. 'Risk Factors for Biopsy Reclassification over Time in Men on Active Surveillance for Early Stage Prostate Cancer', *J Urol*, 204: 1216-21. | Retrospective study |
| 43 | Luzzago, S., M. Catellani, E. Di Trapani, G. Cozzi, F. A. Mistretta, R. Bianchi, P. Pricolo, A. Conti, E. Ancona, N. Piacentini, S. Alessi, G. Renne, M. Ferro, D. V. Matei, G. Musi, B. Alicja Jereczek-Fossa, G. Petralia, and O. de Cobelli. 2020. 'Confirmatory multiparametric magnetic resonance imaging at recruitment confers prolonged stay in active surveillance and decreases the rate of upgrading at follow-up', *Prostate Cancer Prostatic Dis*, 23: 94-101. | Retrospective study |
| 44 | Maggi, M., J. E. Cowan, V. Fasulo, S. L. Washington, 3rd, P. E. Lonergan, A. Sciarra, H. G. Nguyen, and P. R. Carroll. 2020. 'The Long-Term Risks of Metastases in Men on Active Surveillance for Early Stage Prostate Cancer', *J Urol*, 204: 1222-28. | Retrospective study |
| 45 | Mahal, A. R., S. Butler, I. Franco, V. Muralidhar, D. Larios, L. R. G. Pike, S. G. Zhao, N. N. Sanford, R. T. Dess, F. Y. Feng, A. V. D'Amico, D. E. Spratt, J. B. Yu, P. L. Nguyen, T. R. Rebbeck, and B. A. Mahal. 2019. 'Conservative management of low-risk prostate cancer among young versus older men in the United States: Trends and outcomes from a novel national database', *Cancer*, 125: 3338-46. | Retrospective study |
| 46 | Mallya, A., V. Senguttuvan-Karthikeyan, A. Sivaraman, E. Barret, M. Galiano, N. Cathala, A. Mombet, D. Prapotnich, R. Sanchez-Salas, and X. Cathelineau. 2019. 'Immediate versus delayed prostatectomy and the fate of patients who progress to a higher risk disease on active surveillance', *Actas urologicas espanolas*, 43(6): 324-30. | Retrospective study |
| 47 | Matthes, K. L., G. Pestoni, D. Korol, M. Van Hemelrijck, and S. Rohrmann. 2018. 'The risk of prostate cancer mortality and cardiovascular mortality of nonmetastatic prostate cancer patients: A population-based retrospective cohort study', *Urologic Oncology: Seminars and Original Investigations*, 36(6): 309.e15-09.e23. | Retrospective study |
| 48 | Merrick, G. S., A. Tennant, R. Fiano, A. Bennett, R. Anderson, R. Galbreath, W. M. Butler, and E. Adamovich. 2020. 'Active surveillance outcomes in prostate cancer patients: the use of transperineal template-guided mapping biopsy for patient selection', *World J Urol*, 38: 361-69. | Updated data with larger cohort in Merrick, 2021 |
| 49 | Meyer, A. R., M. Mamawala, J. S. Winoker, P. Landis, J. I. Epstein, K. J. Macura, M. E. Allaf, A. W. Partin, C. P. Pavlovich, and M. A. Gorin. 2021. 'Transperineal Prostate Biopsy Improves the Detection of Clinically Significant Prostate Cancer among Men on Active Surveillance', *J Urol*, 205: 1069-74. | Retrospective study |
| 50 | Moon, D. H., R. S. Basak, D. S. Usinger, G. A. Dickerson, D. E. Morris, M. Perman, M. Lim, T. Wibbelsman, J. Chang, Z. Crawford, J. R. Broughman, P. A. Godley, and R. C. Chen. 2019. 'Patient-reported Quality of Life Following Stereotactic Body Radiotherapy and Conventionally Fractionated External Beam Radiotherapy Compared with Active Surveillance Among Men with Localized Prostate Cancer', *Eur Urol*, 76: 391-97. | Clinical outcomes of relevance not reported |
| 51 | Nguyen-Nielsen, M., H. Moller, A. Tjonneland, and M. Borre. 2020. 'Patient-reported outcome measures after treatment for prostate cancer: Results from the Danish Prostate Cancer Registry (DAPROCAdata)', *Cancer Epidemiology*, 64 (no pagination). | Clinical outcomes of relevance not reported |
| 52 | Nyame, Y. A., L. Wilkins, D. J. Greene, V. Ganesan, C. Dai, N. Almassi, A. J. Stephenson, M. Gong, R. Berglund, and E. A. Klein. 2019. 'Assessing the relationship between statin use and oncologic outcomes among men electing active surveillance for localized prostate cancer', *Prostate Cancer Prostatic Dis*, 22: 617-23. | Retrospective study |
| 53 | Olivier, J., V. Kasivisvanathan, E. Drumez, J. C. Fantoni, X. Leroy, P. Puech, and A. Villers. 2019. 'Low-risk prostate cancer selected for active surveillance with negative MRI at entry: can repeat biopsies at 1 year be avoided? A pilot study', *World journal of urology*, 37(2): 253-59. | Pilot study, follow-up <5 years |
| 54 | Parikh, R. B., K. W. Robinson, S. Chhatre, E. Medvedeva, J. P. Cashy, S. Veera, J. M. Bauml, T. Fojo, A. S. Navathe, S. B. Malkowicz, R. Mamtani, and R. Jayadevappa. 2020. 'Comparison by Race of Conservative Management for Low-Risk and Intermediate-Risk Prostate Cancers in Veterans from 2004 to 2018', *JAMA Network Open*, (no pagination). | Retrospective study |
| 55 | Percot, M., G. Robert, F. Bladou, J. M. Ferriere, H. Bensadoun, J. C. Bernhard, E. Alezra, G. Capon, C. Senechal, G. Gourtaud, L. Brureau, V. Roux, P. Blanchet, and R. Eyraud. 2020. 'Active surveillance in prostate cancer is possible for Afro-Caribbean population: Comparison of oncological outcomes with a Caucasian cohort', *Progres en urologie : journal de l'Association francaise d'urologie et de la Societe francaise d'urologie*, 30(10): 532-40. | Retrospective study |
| 56 | Ploussard, G., J. B. Beauval, M. Lesourd, C. Almeras, J. Assoun, R. Aziza, J. R. Gautier, G. Loison, D. Portalez, A. Salin, C. Tollon, M. Soulie, B. Malavaud, and M. Roumiguie. 2020. 'Performance of systematic, MRI-targeted biopsies alone or in combination for the prediction of unfavourable disease in MRI-positive low-risk prostate cancer patients eligible for active surveillance', *World journal of urology*, 38(3): 663-71. | Not an active surveillance study |
| 57 | Ploussard, G., J. B. Beauval, M. Lesourd, C. Manceau, C. Almeras, R. Aziza, J. R. Gautier, G. Loison, D. Portalez, A. Salin, C. Tollon, M. Soulie, B. Malavaud, and M. Roumiguie. 2020. 'Active surveillance eligibility of MRI-positive patients with grade group 2 prostate cancer: a pathological study', *World journal of urology*, 38(7): 1735-40. | Not an active surveillance study |
| 58 | Porreca, A., F. Del Giudice, M. Giampaoli, D. D'Agostino, D. Romagnoli, P. Corsi, A. Del Rosso, M. Maggi, B. I. Chung, M. Ferro, O. de Cobelli, G. Lucarelli, R. Schiavina, E. De Berardinis, A. Sciarra, and G. M. Busetto. 2020. 'Adding systematic biopsy to magnetic resonance ultrasound fusion targeted biopsy of the prostate in men with previous negative biopsy or enrolled in active surveillance programs: A prospective single center, randomized study', *Medicine (Baltimore)*, 99: e22059. | Not an active surveillance study |
| 59 | Raichi, A., G. Marcq, J. C. Fantoni, P. Puech, A. Villers, and A. Ouzzane. 2018. 'Active surveillance in prostate cancer: Assessment of MRI in the selection and follow-up of patients. [French]', *Progres en urologie : journal de l'Association francaise d'urologie et de la Societe francaise d'urologie*, 28(8-9): 416-24. | Article in French, retrospective study |
| 60 | Roscigno, M., A. Stabile, G. Lughezzani, P. Pepe, A. B. Galosi, A. Naselli, R. Naspro, M. Nicolai, G. La Croce, M. Aljoulani, G. Perugini, G. Guazzoni, F. Montorsi, L. Balzarini, S. Sironi, and L. F. Da Pozzo. 2020. 'The Use of Multiparametric Magnetic Resonance Imaging for Follow-up of Patients Included in Active Surveillance Protocol. Can PSA Density Discriminate Patients at Different Risk of Reclassification?', *Clin Genitourin Cancer*, 18: e698-e704. | Retrospective study |
| 61 | Salari, K., D. Kuppermann, M. A. Preston, D. M. Dahl, G. W. Barrisford, J. A. Efstathiou, M. L. Blute, D. Vesprini, A. Loblaw, A. L. Zietman, L. Klotz, and A. S. Feldman. 2019. 'Active Surveillance of Prostate Cancer is a Viable Option for Men Younger than 60 Years', *J Urol*, 201: 721-27. | Retrospective study |
| 62 | Schenk, J. M., L. F. Newcomb, Y. Zheng, A. V. Faino, K. Zhu, Y. A. Nyame, J. D. Brooks, P. R. Carroll, M. R. Cooperberg, A. Dash, C. P. Filson, M. E. Gleave, M. Liss, F. M. Martin, T. M. Morgan, P. S. Nelson, I. M. Thompson, A. A. Wagner, and D. W. Lin. 2020. 'African American Race is Not Associated with Risk of Reclassification during Active Surveillance: Results from the Canary Prostate Cancer Active Surveillance Study', *J Urol*, 203: 727-33. | Sub-group analysis of Canary PASS cohort |
| 63 | Shelton, T. M., J. W. Greenberg, J. L. Silberstein, and L. S. Krane. 2020. 'Hematologic parameters are not predictors of upgrading or treatment in a racially diverse prospective study of men with prostate cancer on active surveillance', *Aging Male*, 23: 1400-08. | <100 patients |
| 64 | Shin, D. W., K. Han, H. S. Park, S. P. Lee, S. H. Park, and J. Park. 2020. 'Risk of Ischemic Heart Disease and Stroke in Prostate Cancer Survivors: A Nationwide Study in South Korea', *Sci Rep*, 10: 10313. | Clinical outcomes of relevance not reported |
| 65 | Soeterik, T. F. W., H. H. E. van Melick, L. M. Dijksman, D. H. Biesma, J. A. Witjes, and J. A. van Basten. 2019. 'Follow-up in Active Surveillance for Prostate Cancer: Strict Protocol Adherence Remains Important for PRIAS-ineligible Patients', *Eur Urol Oncol*, 2: 483-89. | Retrospective study |
| 66 | Stavrinides, V., F. Giganti, B. Trock, S. Punwani, C. Allen, A. Kirkham, A. Freeman, A. Haider, R. Ball, N. McCartan, H. Whitaker, C. Orczyk, M. Emberton, and C. M. Moore. 2020. 'Five-year Outcomes of Magnetic Resonance Imaging-based Active Surveillance for Prostate Cancer: A Large Cohort Study[Formula presented]', *European Urology*, 78(3): 443-51. | Retrospective study |
| 67 | Sugano, D., A. L. Abreu, G. E. Cacciamani, I. S. Gill, and A. H. Lebastchi. 2021. 'Focal Therapy for Low-Risk Prostate Cancer Opinion: No', *Journal of Endourology*, 35(9): 1284-87. | Not an active surveillance study |
| 68 | Talwar, R., B. Friel, S. Mittal, L. Xia, C. Fonshell, J. Danella, B. Jacobs, T. Lanchoney, J. Raman, J. Tomaszewski, E. Trabulsi, A. Reese, E. A. Singer, S. Ginzburg, M. Smaldone, R. Uzzo, P. Mucksavage, T. J. Guzzo, and D. J. Lee. 2020. 'Concordance of confirmatory prostate biopsy in active surveillance with national guidelines: An analysis from the multi-institutional PURC cohort', *Urol Oncol*, 38: 846.e17-46.e22. | Retrospective study |
| 69 | Thurtle, D., T. Barrett, V. Thankappan-Nair, B. Koo, A. Warren, C. Kastner, K. Saeb-Parsy, J. Kimberley-Duffell, and V. J. Gnanapragasam. 2018. 'Progression and treatment rates using an active surveillance protocol incorporating image-guided baseline biopsies and multiparametric magnetic resonance imaging monitoring for men with favourable-risk prostate cancer', *BJU Int*, 122: 59-65. | Follow-up <5 years |
| 70 | Tomer, A., D. Rizopoulos, D. Nieboer, F. J. Drost, M. J. Roobol, and E. W. Steyerberg. 2019. 'Personalized Decision Making for Biopsies in Prostate Cancer Active Surveillance Programs', *Medical decision making : an international journal of the Society for Medical Decision Making*, 39(5): 499-508. | Retrospective study |
| 71 | Ullrich, T., C. Arsov, M. Quentin, F. Mones, A. C. Westphalen, D. Mally, A. Hiester, P. Albers, G. Antoch, and L. Schimmöller. 2020. 'Multiparametric magnetic resonance imaging can exclude prostate cancer progression in patients on active surveillance: a retrospective cohort study', *Eur Radiol*, 30: 6042-51. | Retrospective study |
| 72 | Van Hemelrijck, M., X. Ji, J. Helleman, M. J. Roobol, W. van der Linden, D. Nieboer, C. H. Bangma, M. Frydenberg, A. Rannikko, L. S. Lee, V. J. Gnanapragasam, and M. W. Kattan. 2019. 'Reasons for Discontinuing Active Surveillance: Assessment of 21 Centres in 12 Countries in the Movember GAP3 Consortium', *Eur Urol*, 75: 523-31. | Retrospective study |
| 73 | Walker, C. H., K. A. Marchetti, U. Singhal, and T. M. Morgan. 2021. 'Active surveillance for prostate cancer: selection criteria, guidelines, and outcomes', *World journal of urology.*, 02. | Systematic review |
| 74 | Washington, S. L., 3rd, A. S. Baskin, N. Ameli, H. G. Nguyen, A. C. Westphalen, K. Shinohara, and P. R. Carroll. 2020. 'MRI-Based Prostate-Specific Antigen Density Predicts Gleason Score Upgrade in an Active Surveillance Cohort', *AJR Am J Roentgenol*, 214: 574-78. | Retrospective study |
| 75 | Weissbach, L., E. A. Boedefeld, and J. Herden. 2021. 'Active surveillance-much safety, little recruitment: Is it possible to extend the indication for "intermediate-risk" prostate cancer?. [German]', *Urologe*, 60(10): 1304-12. | Article in German, <100 patients |
| 76 | Wiegel, T., P. Albers, D. Bartkowiak, R. Bussar-Maatz, M. Härter, G. Kristiansen, P. Martus, S. Wellek, H. Schmidberger, K. Grozinger, P. Renner, F. Schneider, M. Burmester, and M. Stöckle. 2021. 'Results of a randomized trial of treatment modalities in patients with low or early-intermediate risk prostate cancer (PREFERE trial)', *J Cancer Res Clin Oncol*, 147: 235-42. | Follow-up <5 years |
| 77 | Williams, C., N. R. Khondakar, M. A. Daneshvar, L. P. O'Connor, P. T. Gomella, S. Mehralivand, N. K. Yerram, J. Egan, S. Gurram, A. Rompré-Brodeur, B. R. Webster, J. Owens-Walton, H. Parnes, M. J. Merino, B. J. Wood, P. Choyke, B. Turkbey, and P. A. Pinto. 2021. 'The Risk of Prostate Cancer Progression in Active Surveillance Patients with Bilateral Disease Detected by Combined Magnetic Resonance Imaging-Fusion and Systematic Biopsy', *J Urol*, 206: 1157-65. | Retrospective study |
| 78 | Yerram, N. K., L. Long, L. P. O'Connor, A. Z. Wang, M. Ahdoot, A. H. Lebastchi, S. Gurram, J. Zeng, H. Chalfin, S. A. Harmon, S. Mehralivand, M. J. Merino, H. L. Parnes, P. L. Choyke, J. Shih, B. J. Wood, B. Turkbey, and P. A. Pinto. 2021. 'Magnetic Resonance Imaging-Targeted and Systematic Biopsy for Detection of Grade Progression in Patients on Active Surveillance for Prostate Cancer', *J Urol*, 205: 1352-60. | Follow-up <5 years |

**Table S3b: Reasons for records excluded from autopsy studies (n=12).**

| No. | Article | Reasons for exclusion |
| --- | --- | --- |
| 1 | Al-Saad, N. F. 2020. 'Immunohistochemical Localization of P27 & Cdk2 Tumor Suppressor Genes Expression in Tissues from Prostatic Tumors', *Biochemical and Cellular Archives*, 20(2): 6701-09. | Not an autopsy study |
| 2 | Alsayigh, H., S. M. Ali, T. Al Mahbob, and S. Al Salihy. 2016. 'Molecular detection of human papillomavirus genotype-31 in tissues from patients with prostate cancer and benign prostatic hyperplasia', *International Journal of PharmTech Research*, 9(5): 277-83. | Not an autopsy study |
| 3 | Barchuk, A., R. Tursun-zade, A. Belayev, M. Moore, Y. Komarov, N. Moshina, A. Anttila, J. Nevalainen, A. Auvinen, A. Ryzhov, and A. Znaor. 2021. 'Comparability and validity of cancer registry data in the northwest of Russia', *Acta Oncologica*, 60(10): 1264-71. | Not an autopsy study |
| 4 | Bosland, M. C., O. S. Nettey, A. A. Phillips, C. C. Anunobi, O. Akinloye, I. O. A. Ekanem, I. A. E. Bassey, V. Mehta, V. Macias, T. H. van der Kwast, and A. B. Murphy. 2021. 'Prevalence of prostate cancer at autopsy in Nigeria-A preliminary report', *Prostate*, 81(9): 553-59. | <100 patients |
| 5 | Erinomo, O. O., C. C. Anunobi, and N. O. Orah. 2013. 'Autopsy study of prostatic weight and lesions in LUTH: a 12 month prospective study', *Nigerian quarterly journal of hospital medicine*, 23(2): 85-89. | Covered by Bell systematic review search period |
| 6 | Hitosugi, M., K. Mukaisho, M. Kido, S. Kamimura, S. Furukawa, and H. Sugihara. 2017. 'No change in the prevalence of latent prostate cancer over the last 10 years: a forensic autopsy study in Japan', *Biomed Res*, 38: 307-12. | Data updated in publication by Kido |
| 7 | Jacklin, C., Y. Philippou, S. F. Brewster, and R. J. Bryant. 2021. '“More men die with prostate cancer than because of it” – an old adage that still holds true in the 21^st^ century.', *Cancer Treatment and Research Communications,* 26: 100225. | Systematic review |
| 8 | Siadat, F., J. Sykes, A. R. Zlotta, N. Aldaoud, S. Egawa, D. Pushkar, C. Kuk, R. G. Bristow, R. Montironi, and T. van der Kwast. 2015. 'Not all gleason pattern 4 prostate cancers are created equal: A study of latent prostatic carcinomas in a cystoprostatectomy and autopsy series', *Prostate*, 75: 1277-84. | Uses data from Zlotta 2013 and Mazzucchelli 2009 |
| 9 | Takeshima, Y., M. Suzuki, J. Miyakawa, I. Tsuru, Y. Yamada, M. Nakamura, Y. Sato, T. Kawai, D. Yamada, T. Morikawa, and H. Kume. 2021. 'Latent prostate cancer among Japanese males: a bibliometric study of autopsy reports from 1980-2016', *Jpn J Clin Oncol*, 51: 156-59. | Prostate cancers found at autopsy may have contributed to cause of death |
| 10 | Zlotta, A. R., S. Egawa, D. Pushkar, A. Govorov, T. Kimura, M. Kido, H. Takahashi, C. Kuk, M. Kovylina, N. Aldaoud, N. Fleshner, A. Finelli, L. Klotz, J. Sykes, G. Lockwood, and T. H. van der Kwast. 2013. 'Prevalence of prostate cancer on autopsy: cross-sectional study on unscreened Caucasian and Asian men', *J Natl Cancer Inst*, 105: 1050-8. | Identified in Bell systematic review |
| 11 | Zlotta, A. R., and C. Kuk. 2014. 'Prevalence of prostate cancer across the globe: what can autopsy studies teach us about this peculiar disease?', *Arch Esp Urol*, 67: 400-8. | Systematic review |
| 12 | Zlotta, A. R., S. Egawa, D. Pushkar, A. Govorov, T. Kimura, M. Kido, H. Takahashi, C. Kuk, M. Kovylina, N. Aldaoud, N. Fleshner, A. Finelli, L. Klotz, G. Lockwood, J. Sykes, and Tv Kwast. 2014. 'Prevalence of inflammation and benign prostatic hyperplasia on autopsy in Asian and Caucasian men', *Eur Urol*, 66: 619-22. | Same cohort as Zlotta 2013, identifies rates of BPH and inflammation |

**Table S3c: Reasons for records excluded from reproducibility studies (n=112).**

| No. | Article | Reasons for exclusion |
| --- | --- | --- |
| 1 | Abdollahi, A., A. Meysamie, S. Sheikhbahaei, A. Ahmadi, H. Moradi-Tabriz, M. Bakhshandeh, and H. Hosseinzadeh. 2012. 'Inter/Intra-observer reproducibility of Gleason scoring in prostate adenocarcinoma in Iranian pathologists', *Urology Journal*, 9(2): 486-90. | <10 pathologists |
| 2 | Abdollahi, A., S. Sheikhbahaei, A. Meysamie, M. Bakhshandeh, and H. Hosseinzadeh. 2014. 'Inter-observer reproducibility before and after web-based education in the Gleason grading of the prostate adenocarcinoma among the Iranian pathologists', *Acta Medica Iranica*, 52(5): 370-74. | <10 pathologists |
| 3 | Al Nemer, A. M., T. Elsharkawy, M. Elshawarby, D. Al-Tamimi, H. Kussaibi, and A. Ahmed. 2017. 'The updated grading system of prostate carcinoma: an inter-observer agreement study among general pathologists in an academic practice', *Apmis*, 125(11): 957-61. | <10 pathologists |
| 4 | Al-Hussain, T. O., M. S. Nagar, and J. I. Epstein. 2012. 'Gleason pattern 5 is frequently underdiagnosed on prostate needle-core biopsy', *Urology*, 79: 178-81. | Reproducibility of Gleason pattern 5 only |
| 5 | Al-Maghrabi, J. A., N. A. Bakshi, and H. M. A. Farsi. 2013. 'Gleason grading of prostate cancer in needle core biopsies: A comparison of general and urologic pathologists', *Annals of Saudi Medicine*, 33(1): 40-44. | <10 pathologists |
| 6 | Al-Rikabi, A. C., and H. Alkhalidi. 2014. 'Interobserver variations in reporting of prostatic adenocarcinoma using core biopsy specimens: A retrospective study from a tertiary referral hospital in Saudi Arabia', *Eastern Mediterranean Health Journal*, 20(9): 578-81. | <10 pathologists |
| 7 | Aldaoud, N., A. Hallak, N. Abdo, S. Al Bashir, N. Marji, and A. Graboski-Bauer. 2020. 'Interobserver Variability in the Diagnosis of High-Grade Prostatic Intraepithelial Neoplasia in a Tertiary Hospital in Northern Jordan', *Clinical Pathology*, 13. | <10 pathologists |
| 8 | Algaba Arrea, F., A. Chivite de Leon, J. M. Santaularia Segura, and A. Oliver Samper. 2004. 'Evidence of the radical prostatectomy Gleason score in the biopsy Gleason score. [Spanish]', *Actas urologicas espanolas*, 28(1): 21-26. | Non-English manuscript |
| 9 | Allam, C. K., D. G. Bostwick, J. A. Hayes, M. P. Upton, G. G. Wade, G. F. Domanowski, M. A. Klein, E. A. Boling, and M. M. Stilmant. 1996. 'Interobserver variability in the diagnosis of high-grade prostatic intraepithelial neoplasia and adenocarcinoma', *Modern Pathology*, 9(7): 742-51. | <10 pathologists |
| 10 | Allsbrook Jr, W. C., K. A. Mangold, M. H. Johnson, R. B. Lane, C. G. Lane, M. B. Amin, D. G. Bostwick, P. A. Humphrey, E. C. Jones, V. E. Reuter, W. Sakr, I. A. Sesterhenn, P. Troncoso, T. M. Wheeler, and J. I. Epstein. 2001. 'Interobserver reproducibility of Gleason grading of prostatic carcinoma: Urologic pathologists', *Human Pathology*, 32(1): 74-80. | At high risk of bias, lack of sample representativeness |
| 11 | Allsbrook Jr, W. C., K. A. Mangold, M. H. Johnson, R. B. Lane, C. G. Lane, and J. I. Epstein. 2001. 'Interobserver reproducibility of Gleason grading of prostatic carcinoma: General pathologists', *Human Pathology*, 32(1): 81-88. | At high risk of bias, lack of sample representativeness |
| 12 | Arista-Nasr, J., E. Cortes, C. Keirns, A. Hatchett, and A. Loria. 1996. 'Diagnostic concordance in biopsies of deceptive prostatic carcinoma', *Revista de investigacion clinica; organo del Hospital de Enfermedades de la Nutricion*, 48(4): 289-96. | Does not use Gleason scoring |
| 13 | Azakpa, A. L., F. F. Priuli, E. Ndayake, E. Ganhouingnon, I. Gonzalez-Rodilla, M. P. Tchaou, and T. Zanin. 2021. 'Telepathology Practice in Cancer Diagnosis in Saint Jean de Dieu Hospital - Tanguieta, Benin', *Arch Pathol Lab Med*, 145: 871-76. | Not a reproducibility study |
| 14 | Bain, G. O., M. Koch, and J. Hanson. 1982. 'Feasibility of Grading Prostatic Carcinomas', *Arch Pathol Lab Med,* 106: 265-267. | <10 pathologists |
| 15 | Berner, A., S. Harvei, and F. J. Skjorten. 1999. 'Follow-up of localized prostate cancer, with emphasis on previous undiagnosed incidental cancer', *BJU International*, 83(1): 47-52. | <10 pathologists |
| 16 | Bori, R., F. Salamon, C. Moczar, and G. Cserni. 2013. 'Interobserver reproducibility of Gleason grading in prostate biopsy samples. [Hungarian, English]', *Orvosi Hetilap*, 154(31): 1219-25. | Non-English manuscript |
| 17 | Bostwick, D. G., and L. Chang. 1999. 'Overdiagnosis of prostatic adenocarcinoma', *Semin Urol Oncol*, 17: 199-205. | <10 pathologists |
| 18 | Bostwick, D. G., and J. Ma. 2007. 'Over-diagnosis of high-grade prostatic intraepithelial neoplasia: A prospective study of 251 cases', *BJU International*, 100(5): 1036-39. | <10 pathologists |
| 19 | Bova, G. Steven, G. Parmigiani, J. I. Epstein, T. Wheeler, N. R. Mucci, and M. A. Rubin. 2001. 'Web-based tissue microarray image data analysis: Initial validation testing through prostate cancer gleason grading', *Human Pathology*, 32(4): 417-27. | <10 pathologists |
| 20 | Bryant, R. J., A. J. Schmitt, I. S. Roberts, P. S. Gill, L. Browning, S. F. Brewster, F. C. Hamdy, and C. Verrill. 2015. 'Variation between specialist uropatholgists in reporting extraprostatic extension after radical prostatectomy', *J Clin Pathol*, 68: 465-72. | Reproducibility of extra-prostatic extension |
| 21 | Burchardt, M., R. Engers, M. Muller, T. Burchardt, R. Willers, J. I. Epstein, R. Ackermann, H. E. Gabbert, A. De La Taille, and M. A. Rubin. 2008. 'Interobserver reproducibility of Gleason grading: Evaluation using prostate cancer tissue microarrays', *Journal of Cancer Research and Clinical Oncology*, 134(10): 1071-78. | At high risk of bias, lack of sample representativeness |
| 22 | Cahill, L. C., Y. Wu, T. Yoshitake, C. Ponchiardi, M. G. Giacomelli, A. A. Wagner, S. Rosen, and J. G. Fujimoto. 2020. 'Nonlinear microscopy for detection of prostate cancer: analysis of sensitivity and specificity in radical prostatectomies', *Modern Pathology*, 33(5): 916-23. | Does not use Gleason scoring |
| 23 | Carmona Echeverria, L. M., A. Haider, A. Freeman, U. Stopka-Farooqui, A. Rosenfeld, B. S. Simpson, Y. Hu, D. Hawkes, H. Pye, S. Heavey, V. Stavrinides, J. M. Norris, A. E. Bosaily, C. Cardona Barrena, S. Bott, L. Brown, N. Burns-Cox, T. Dudderidge, A. Henderson, R. Hindley, R. Kaplan, A. Kirkham, R. Oldroyd, M. Ghei, R. Persad, S. Punwani, D. Rosario, I. Shergill, M. Winkler, H. U. Ahmed, M. Emberton, and H. C. Whitaker. 2020. 'A critical evaluation of visual proportion of Gleason 4 and maximum cancer core length quantified by histopathologists', *Sci Rep*, 10: 17177. | <10 pathologists |
| 24 | Chen, S. D., J. L. Fava, and A. Amin. 2016. 'Gleason grading challenges in the diagnosis of prostate adenocarcinoma: experience of a single institution', *Virchows Archiv*, 468(2): 213-18. | <10 pathologists |
| 25 | Coard, K. C., and V. L. Freeman. 2004. 'Gleason grading of prostate cancer: level of concordance between pathologists at the University Hospital of the West Indies', *Am J Clin Pathol*, 122: 373-6. | <10 pathologists |
| 26 | De Las Morenas, A., M. B. Siroky, J. Merriam, and M. M. Stilmant. 1988. 'Prostatic adenocarcinoma: Reproducibility and correlation with clinical stages of four grading systems', *Human Pathology*, 19(5): 595-97. | <10 pathologists |
| 27 | De Marzo, A. M., E. A. Platz, J. I. Epstein, T. Ali, A. Billis, T. Y. Chan, L. Cheng, M. Datta, L. Egevad, D. Ertoy-Baydar, X. Farre, S. W. Fine, K. A. Iczkowski, M. Ittmann, B. S. Knudsen, M. Loda, A. Lopez-Beltran, C. Magi-Galluzzi, G. Mikuz, R. Montironi, E. Pikarsky, G. Pizov, M. A. Rubin, H. Samaratunga, T. Sebo, I. A. Sesterhenn, R. B. Shah, S. Signoretti, J. Simko, G. Thomas, P. Troncoso, T. T. Tsuzuki, G. J. van Leenders, X. J. Yang, M. Zhou, W. D. Figg, A. Hoque, and M. S. Lucia. 2006. 'A working group classification of focal prostate atrophy lesions', *Am J Surg Pathol*, 30: 1281-91. | Does not use Gleason scoring |
| 28 | Dere, Y., I. Çelik Ö, S. Y. Çelik, S. Ekmekçi, G. Evcim, F. Pehlivan, A. Ağalar, H. Deliktaş, and N. Çulhacı. 2020. 'A grading dilemma; Gleason scoring system: Are we sufficiently compatible? A multi center study', *Indian J Pathol Microbiol*, 63: S25-s29. | <10 pathologists |
| 29 | Di Loreto, C., B. Fitzpatrick, S. Underhill, D. H. Kim, H. E. Dytch, H. Galera-Davidson, and M. Bibbo. 1991. 'Correlation between visual clues, objective architectural features, and interobserver agreement in prostate cancer', *American Journal of Clinical Pathology*, 96(1): 70-75. | <10 pathologists |
| 30 | Egevad, L., A. S. Ahmad, F. Algaba, D. M. Berney, L. Boccon-Gibod, E. Comperat, A. J. Evans, D. Griffiths, R. Grobholz, G. Kristiansen, C. Langner, A. Lopez-Beltran, R. Montironi, S. Moss, P. Oliveira, B. Vainer, M. Varma, and P. Camparo. 2013. 'Standardization of Gleason grading among 337 European pathologists', *Histopathology*, 62(2): 247-56. | At high risk of bias, lack of sample representativeness |
| 31 | Egevad, L., B. Delahunt, D. M. Berney, D. G. Bostwick, J. Cheville, E. Comperat, A. J. Evans, S. W. Fine, D. J. Grignon, P. A. Humphrey, J. Hornblad, K. A. Iczkowski, J. G. Kench, G. Kristiansen, K. R. M. Leite, C. Magi-Galluzzi, J. K. McKenney, J. Oxley, C. C. Pan, H. Samaratunga, J. R. Srigley, H. Takahashi, L. D. True, T. Tsuzuki, T. van der Kwast, M. Varma, M. Zhou, and M. Clements. 2018. 'Utility of Pathology Imagebase for standardisation of prostate cancer grading', *Histopathology*, 73(1): 8-18. | At high risk of bias, lack of sample representativeness |
| 32 | Egevad, L., B. Delahunt, H. Samaratunga, T. Tsuzuki, H. Olsson, P. Strom, C. Lindskog, T. Hakkinen, K. Kartasalo, M. Eklund, and P. Ruusuvuori. 2021. 'Interobserver reproducibility of perineural invasion of prostatic adenocarcinoma in needle biopsies', *Virchows Archiv*, 478(6): 1109-16. | <10 pathologists |
| 33 | Epstein, J. I., D. J. Grignon, P. A. Humphrey, J. E. McNeal, I. A. Sesterhenn, P. Troncoso, and T. M. Wheeler. 1995. 'Interobserver reproducibility in the diagnosis of prostatic intraepithelial neoplasia', *American Journal of Surgical Pathology*, 19(8): 873-86. | <10 pathologists |
| 34 | Flood, T. A., N. Schieda, D. T. Keefe, R. H. Breau, C. Morash, K. Hogan, E. C. Belanger, K. T. Mai, and S. J. Robertson. 2016. 'Utility of Gleason pattern 4 morphologies detected on transrectal ultrasound (TRUS)-guided biopsies for prediction of upgrading or upstaging in Gleason score 3 + 4 = 7 prostate cancer', *Virchows Arch*, 469: 313-9. | <10 pathologists |
| 35 | Freeman, V. L., K. C. Coard, E. Wojcik, and R. Durazo-Arvizu. 2004. 'Use of the Gleason system in international comparisons of prostatic adenocarcinomas in blacks', *Prostate*, 58: 169-73. | <10 pathologists |
| 36 | Fukagai, T., T. Namiki, H. Namiki, R. G. Carlile, M. Shimada, and H. Yoshida. 2001. 'Discrepancies between Gleason scores of needle biopsy and radical prostatectomy specimens', *Pathology International*, 51(5): 364-70. | <10 pathologists |
| 37 | Gallee, M. P., F. J. Ten Kate, P. G. Mulder, J. H. Blom, and R. O. van der Heul. 1990. 'Histological grading of prostatic carcinoma in prostatectomy specimens. Comparison of prognostic accuracy of five grading systems', *Br J Urol*, 65: 368-75. | <10 pathologists, comparison of grading systems |
| 38 | Giesen, R. J., A. L. Huynen, J. J. de la Rosette, H. E. Schaafsma, M. P. van Iersel, R. G. Aarnink, F. M. Debruyne, and H. Wijkstra. 1994. 'The reliability of computer analysis of ultrasonographic prostate images: the influence of inconsistent histopathology', *Ultrasound Med Biol*, 20: 871-6. | Deep learning study, 1 pathologist used for reference |
| 39 | Glaessgen, A., H. Hamberg, C. G. Pihl, B. Sundelin, B. Nilsson, and L. Egevad. 2002. 'Interobserver reproducibility of percent Gleason grade 4/5 in total prostatectomy specimens', *J Urol*, 168: 2006-10. | <10 pathologists |
| 40 | Glaessgen, A., H. Hamberg, C. G. Pihl, B. Sundelin, B. Nilsson, and L. Egevad. 2004. 'Interobserver reproducibility of percent Gleason grade 4/5 in prostate biopsies', *Journal of Urology*, 171(2 I): 664-67. | <10 pathologists |
| 41 | Glaessgen, A., H. Hamberg, C. G. Pihl, B. Sundelin, B. Nilsson, and L. Egevad. 2004. 'Interobserver reproducibility of modified Gleason score in radical prostatectomy specimens', *Virchows Archiv*, 445(1): 17-21. | <10 pathologists |
| 42 | Goodman, M., K. C. Ward, A. O. Osunkoya, M. W. Datta, D. Luthringer, A. N. Young, K. Marks, V. Cohen, J. C. Kennedy, M. J. Haber, and M. B. Amin. 2012. 'Frequency and determinants of disagreement and error in gleason scores: A population-based study of prostate cancer', *Prostate*, 72(13): 1389-98. | <10 pathologists |
| 43 | Grasso, A. A., G. Cozzi, C. Palumbo, G. Albo, and B. Rocco. 2014. 'Concordance between biopsy and radical prostatectomy specimen Gleason score in internal and external pathology facilities', *Anticancer Res*, 34: 5585-8. | <10 pathologists |
| 44 | Harbias, A., E. Salmo, and A. Crump. 2017. 'Implications of Observer Variation in Gleason Scoring of Prostate Cancer on Clinical Management: A Collaborative Audit', *The Gulf journal of oncology*, 1(25): 41-45. | <10 pathologists |
| 45 | He, H., and G. Wang. 2016. 'Reproducibility of Gleason scores in prostate cancer. [Chinese]', *Zhonghua nan ke xue = National journal of andrology*, 22(1): 37-41. | Non-English manuscript |
| 46 | Helpap, B., G. Kristiansen, M. Beer, J. Köllermann, U. Oehler, A. Pogrebniak, and Ch Fellbaum. 2012. 'Improving the reproducibility of the Gleason scores in small foci of prostate cancer--suggestion of diagnostic criteria for glandular fusion', *Pathol Oncol Res*, 18: 615-21. | <10 pathologists |
| 47 | Hoekstra, R. J., W. J. H. Goossens, A. Beulens, H. van Herk, B. M. Hoevenaars, J. de Baaij, D. M. Somford, J. P. M. Sedelaar, J. P. A. van Basten, and H. J. E. J. Vrijhof. 2021. 'Reassessment of Prostate Biopsy Specimens for Patients Referred for Robot-assisted Radical Prostatectomy Rarely Influences Surgical Planning', *European Urology Open Science*, 28: 36-42. | <10 pathologists |
| 48 | Iczkowski, K. A., L. Egevad, J. Ma, N. Harding-Jackson, F. Algaba, A. Billis, P. Camparo, L. Cheng, D. Clouston, E. M. Comperat, M. W. Datta, A. G. Evans, D. F. Griffiths, C. C. Guo, S. Hailemariam, W. Huang, P. A. Humphrey, Z. Jiang, H. Kahane, G. Kristiansen, F. G. La Rosa, A. Lopez-Beltran, G. T. MacLennan, C. Magi-Galluzzi, J. Merrimen, R. Montironi, A. O. Osunkoya, M. M. Picken, N. Rao, R. B. Shah, J. H. Shanks, S. S. Shen, O. W. Tawfik, L. D. True, T. Van Der Kwast, M. Varma, T. M. Wheeler, D. L. Zynger, N. Sahr, and D. G. Bostwick. 2014. 'Intraductal carcinoma of the prostate: Interobserver reproducibility survey of 39 urologic pathologists', *Annals of Diagnostic Pathology*, 18(6): 333-42. | Does not use Gleason scoring |
| 49 | Jara-Lazaro, A. R., A. A. Thike, and P. H. Tan. 2010. 'Diagnostic issues in second opinion consultations in prostate pathology', *Pathology*, 42: 6-14. | <10 pathologists |
| 50 | Jorgensen, T., K. Yogesan, F. Skjorten, A. Berner, K. J. Tveter, and H. E. Danielsen. 1995. 'Histopathological grading and DNA ploidy as prognostic markers in metastatic prostatic cancer', *British Journal of Cancer*, 71(5): 1055-60. | <10 pathologists |
| 51 | Junker, D., T. R. Herrmann, M. Bader, J. Bektic, G. Henkel, S. Kruck, M. Sandbichler, D. Schilling, G. Schafer, and U. Nagele. 2016. 'Evaluation of the 'Prostate Interdisciplinary Communication and Mapping Algorithm for Biopsy and Pathology' (PIC-MABP)', *World journal of urology*, 34(2): 245-52. | Reproducibility of MRI findings |
| 52 | Kiss, F., G. Lakner, M. Csellar, P. Nagy, A. Toth, and G. Vittay. 1997. 'Reproducibility of malignancy grading in prostatic cancers using the Gleason-Bockling system. [Hungarian]', *Orvosi Hetilap*, 138(19): 1195-99. | Non-English manuscript |
| 53 | Kronz, J. D., M. A. Silberman, W. C. Allsbrook, and J. I. Epstein. 2000. 'A web-based tutorial improves practicing pathologists' Gleason grading of images of prostate carcinoma specimens obtained by needle biopsy: validation of a new medical education paradigm', *Cancer*, 89: 1818-23. | Limited information reported regarding reproducibility. Interobserver reproducibility not reported. |
| 54 | Kuroiwa, K., T. Shiraishi, O. Ogawa, M. Usami, Y. Hirao, and S. Naito. 2010. 'Discrepancy Between Local and Central Pathological Review of Radical Prostatectomy Specimens', *Journal of Urology*, 183(3): 952-57. | <10 pathologists |
| 55 | Kweldam, C. F., D. Nieboer, F. Algaba, M. B. Amin, D. M. Berney, A. Billis, D. G. Bostwick, L. Bubendorf, L. Cheng, E. Comperat, B. Delahunt, L. Egevad, A. J. Evans, D. E. Hansel, P. A. Humphrey, G. Kristiansen, T. H. van der Kwast, C. Magi-Galluzzi, R. Montironi, G. J. Netto, H. Samaratunga, J. R. Srigley, P. H. Tan, M. Varma, M. Zhou, and G. J. L. H. van Leenders. 2016. 'Gleason grade 4 prostate adenocarcinoma patterns: an interobserver agreement study among genitourinary pathologists', *Histopathology*, 69(3): 441-49. | At high risk of bias, lack of sample representativeness |
| 56 | Li, J., W. Li, A. Sisk, H. Ye, W. D. Wallace, W. Speier, and C. W. Arnold. 2021. 'A multi-resolution model for histopathology image classification and localization with multiple instance learning', *Computers in Biology and Medicine*, 131 (no pagination). | Not a reproducibility study |
| 57 | Lucia, M. S., D. G. Bostwick, M. C. Somerville, I. L. Fowler, and R. S. Rittmaster. 2013. 'Comparison of classic and International Society of Urological Pathology 2005 modified Gleason grading using needle biopsies from the Reduction by Dutasteride of prostate Cancer Events (REDUCE) trial', *Archives of Pathology and Laboratory Medicine*, 137(12): 1740-46. | <10 pathologists |
| 58 | Lundin, M., J. Lundin, P. Martikainen, T. Tammela, H. Helin, T. Van Der Kwast, and J. Isola. 2005. 'Web-based virtual microscopy in teaching and standardizing Gleason grading', *Human Pathology*, 36(4): 381-86. | <10 pathologists |
| 59 | Mansouri, N., I. Msakni, F. Gargouri, R. Khiari, A. Bouziani, and B. Laabidi. 2018. 'Evaluation of concordance of Gleason score between prostate biopsy and radical prostatectomy', *Tunis Med*, 96: 430-36. | Non-English manuscript |
| 60 | McGarry, S. D., J. D. Bukowy, K. A. Iczkowski, A. K. Lowman, M. Brehler, S. Bobholz, A. Nencka, A. Barrington, K. Jacobsohn, J. Unteriner, P. Duvnjak, M. Griffin, M. Hohenwalter, T. Keuter, W. Huang, T. Antic, G. Paner, W. Palangmonthip, A. Banerjee, and P. S. LaViolette. 2020. 'Radio-pathomic mapping model generated using annotations from five pathologists reliably distinguishes high-grade prostate cancer', *Journal of Medical Imaging*, 7(5) (no pagination). | Interobserver reproducibility of annotations rather than diagnostic categories |
| 61 | McLean, M., J. Srigley, D. Banerjee, P. Warde, and Y. Hao. 1997. 'Interobserver variation in prostate cancer Gleason scoring: Are there implications for the design of clinical trials and treatment strategies?', *Clinical Oncology*, 9(4): 222-25. | <10 pathologists |
| 62 | Melia, J., R. Moseley, R. Y. Ball, D. F. Griffiths, K. Grigor, P. Harnden, M. Jarmulowicz, L. J. McWilliam, R. Montironi, M. Waller, S. Moss, and M. C. Parkinson. 2006. 'A UK-based investigation of inter- and intra-observer reproducibility of Gleason grading of prostatic biopsies', *Histopathology*, 48: 644-54. | <10 pathologists |
| 63 | Meliti, A., E. Sadimin, M. Diolombi, F. Khani, and J. I. Epstein. 2017. 'Accuracy of Grading Gleason Score 7 Prostatic Adenocarcinoma on Needle Biopsy: Influence of Percent Pattern 4 and Other Histological Factors', *Prostate*, 77(6): 681-85. | <10 pathologists |
| 64 | Mihatsch, M. J., H. Ohnacker, M. Oberholzer, H. P. Spichtin, T. Eichenberger, E. Perret, and J. Torhorst. 1983. '[How reliable is cancer diagnosis with needle biopsy of the prostate?]', *Urologe A*, 22: 202-7. | Non-English manuscript |
| 65 | Mikami, Y., T. Manabe, J. I. Epstein, T. Shiraishi, M. Furusato, T. Tsuzuki, Y. Matsuno, and H. Sasano. 2003. 'Accuracy of Gleason grading by practicing pathologists and the impact of education on improving agreement', *Human Pathology*, 34(7): 658-65. | At high risk of bias, lack of sample representativeness |
| 66 | Montironi, R., D. Thompson, M. Scarpelli, H. G. Bartels, P. W. Hamilton, V. D. da Silva, W. A. Sakr, B. Weyn, A. van Daele, and P. H. Bartels. 2002. 'Transcontinental communication and quantitative digital histopathology via the Internet; with special reference to prostate neoplasia', *J Clin Pathol*, 55: 452-60. | <10 pathologists |
| 67 | Montironi, R., R. Y. Ball, D. F. Griffiths, K. Grigor, P. M. Harnden, M. Jarmulowicz, L. J. McWilliam, R. P. Moseley, M. C. Parkinson, A. Santinelli, S. M. Moss, and J. W. Melia. 2008. 'Bayesian belief network for the Gleason patterns in prostatic adenocarcinoma: development of a diagnostic decision support system for educational purposes', *Anal Quant Cytol Histol*, 30: 8-15. | <10 pathologists |
| 68 | Mortezavi, A., E. X. Keller, C. Poyet, T. Hermanns, K. Saba, M. Randazzo, C. D. Fankhauser, P. J. Wild, H. Moch, T. Sulser, and D. Eberli. 2016. 'Clinical impact of prostate biopsy undergrading in an academic and community setting', *World journal of urology*, 34(10): 1481-90. | <10 pathologists |
| 69 | Nagpal, K., D. Foote, Y. Liu, P. H. C. Chen, E. Wulczyn, F. Tan, N. Olson, J. L. Smith, A. Mohtashamian, J. H. Wren, G. S. Corrado, R. MacDonald, L. H. Peng, M. B. Amin, A. J. Evans, A. R. Sangoi, C. H. Mermel, J. D. Hipp, and M. C. Stumpe. 2019. 'Development and validation of a deep learning algorithm for improving Gleason scoring of prostate cancer', *npj Digital Medicine*, 2(1) (no pagination). | Limited information reported regarding reproducibility of GG1 lesions |
| 70 | Nagpal, K., D. Foote, F. Tan, Y. Liu, P. C. Chen, D. F. Steiner, N. Manoj, N. Olson, J. L. Smith, A. Mohtashamian, B. Peterson, M. B. Amin, A. J. Evans, J. W. Sweet, C. Cheung, T. van der Kwast, A. R. Sangoi, M. Zhou, R. Allan, P. A. Humphrey, J. D. Hipp, K. Gadepalli, G. S. Corrado, L. H. Peng, M. C. Stumpe, and C. H. Mermel. 2020. 'Development and Validation of a Deep Learning Algorithm for Gleason Grading of Prostate Cancer From Biopsy Specimens', *JAMA Oncol*, 6: 1372-80. | <10 pathologists |
| 71 | Nakai, Y., N. Tanaka, K. Shimada, N. Konishi, M. Miyake, S. Anai, and K. Fujimoto. 2015. 'Review by urological pathologists improves the accuracy of Gleason grading by general pathologists', *BMC Urol*, 15: 70. | <10 pathologists |
| 72 | Netto, G. J., M. Eisenberger, and J. I. Epstein. 2011. 'Interobserver variability in histologic evaluation of radical prostatectomy between central and local pathologists: Findings of TAX 3501 multinational clinical trial', *Urology*, 77(5): 1155-60. | <10 pathologists |
| 73 | Nguyen, P. L., D. Schultz, A. A. Renshaw, R. T. Vollmer, W. R. Welch, K. Cote, and A. V. D'Amico. 2004. 'The impact of pathology review on treatment recommendations for patients with adenocarcinoma of the prostate', *Urologic Oncology: Seminars and Original Investigations*, 22(4): 295-99. | <10 pathologists |
| 74 | Nir, G., S. Hor, D. Karimi, L. Fazli, B. F. Skinnider, P. Tavassoli, D. Turbin, C. F. Villamil, G. Wang, R. S. Wilson, K. A. Iczkowski, M. S. Lucia, P. C. Black, P. Abolmaesumi, S. L. Goldenberg, and S. E. Salcudean. 2018. 'Automatic grading of prostate cancer in digitized histopathology images: Learning from multiple experts', *Medical Image Analysis*, 50: 167-80. | <10 pathologists |
| 75 | Oyama, T., W. C. Allsbrook, Jr., K. Kurokawa, H. Matsuda, A. Segawa, T. Sano, K. Suzuki, and J. I. Epstein. 2005. 'A comparison of interobserver reproducibility of Gleason grading of prostatic carcinoma in Japan and the United States', *Arch Pathol Lab Med*, 129: 1004-10. | At high risk of bias, lack of sample representativeness |
| 76 | Ozkan, T. A., A. T. Eruyar, O. O. Cebeci, O. Memik, L. Ozcan, and I. Kuskonmaz. 2016. 'Interobserver variability in Gleason histological grading of prostate cancer', *Scandinavian Journal of Urology*, 50(6): 420-24. | <10 pathologists |
| 77 | Pantanowitz, L., G. M. Quiroga-Garza, L. Bien, R. Heled, D. Laifenfeld, C. Linhart, J. Sandbank, A. Albrecht Shach, V. Shalev, M. Vecsler, P. Michelow, S. Hazelhurst, and R. Dhir. 2020. 'An artificial intelligence algorithm for prostate cancer diagnosis in whole slide images of core needle biopsies: a blinded clinical validation and deployment study', *The Lancet Digital Health*, 2(8): e407-e16. | <10 pathologists |
| 78 | Persson, J., U. Wilderang, T. Jiborn, P. N. Wiklund, J. E. Damber, J. Hugosson, G. Steineck, E. Haglind, and A. Bjartell. 2014. 'Interobserver variability in the pathological assessment of radical prostatectomy specimens: Findings of the Laparoscopic Prostatectomy Robot Open (LAPPRO) study', *Scandinavian Journal of Urology*, 48(2): 160-67. | <10 pathologists |
| 79 | Puliatti, S., L. Bertoni, G. M. Pirola, P. Azzoni, L. Bevilacqua, A. Eissa, A. Elsherbiny, M. C. Sighinolfi, J. Chester, S. Kaleci, B. Rocco, S. Micali, I. Bagni, L. R. Bonetti, A. Maiorana, J. Malvehy, C. Longo, R. Montironi, G. Bianchi, and G. Pellacani. 2019. 'Ex vivo fluorescence confocal microscopy: the first application for real-time pathological examination of prostatic tissue', *BJU International*, 124(3): 469-76. | Confocal microscopy study, <10 pathologists reviewed histopathological slides |
| 80 | Rao, V., P. Subramanian, A. Sali, S. Menon, and S. Desai. 2021. 'Validation of Whole Slide Imaging for primary surgical pathology diagnosis of prostate biopsies', *Indian Journal of Pathology and Microbiology*, 64(1): 78-83. | <10 pathologists |
| 81 | Rocco, B., M. C. Sighinolfi, M. Sandri, V. Spandri, A. Cimadamore, M. Volavsek, R. Mazzucchelli, A. Lopez-Beltran, A. Eissa, L. Bertoni, P. Azzoni, L. Reggiani Bonetti, A. Maiorana, S. Puliatti, S. Micali, M. Paterlini, A. Iseppi, F. Rocco, G. Pellacani, J. Chester, G. Bianchi, and R. Montironi. 2020. 'Digital Biopsy with Fluorescence Confocal Microscope for Effective Real-time Diagnosis of Prostate Cancer: A Prospective, Comparative Study', *European Urology Oncology.* | <10 pathologists |
| 82 | Rodriguez-Urrego, P. A., A. M. Cronin, H. A. Al-Ahmadie, A. Gopalan, S. K. Tickoo, V. E. Reuter, and S. W. Fine. 2011. 'Interobserver and intraobserver reproducibility in digital and routine microscopic assessment of prostate needle biopsies', *Human Pathology*, 42(1): 68-74. | <10 pathologists |
| 83 | Rousselet, M. C., J. P. Saint-Andre, P. Six, and J. Y. Soret. 1986. 'Reproducibility and prognostic value of Gleason's and Gaeta's histological grades in prostatic carcinoma. [French]', *Annales d'urologie*, 20(5): 317-22. | Non-English manuscript |
| 84 | Ruprecht, O., P. Weisser, B. Bodelle, H. Ackermann, and T. J. Vogl. 2012. 'MRI of the prostate: interobserver agreement compared with histopathologic outcome after radical prostatectomy', *Eur J Radiol*, 81: 456-60. | Reproducibility of MRI findings |
| 85 | Ryu, H. S., M. S. Jin, J. H. Park, S. Lee, J. Cho, S. Oh, T. Y. Kwak, J. Isaacwoo, Y. Mun, S. W. Kim, S. Hwang, S. J. Shin, and H. Chang. 2019. 'Automated gleason scoring and tumor quantification in prostate core needle biopsy images using deep neural networks and its comparison with pathologist-based assessment', *Cancers*, 11(12) (no pagination). | <10 pathologists |
| 86 | Sadimin, E. T., F. Khani, M. Diolombi, A. Meliti, and J. I. Epstein. 2016. 'Interobserver Reproducibility of Percent Gleason Pattern 4 in Prostatic Adenocarcinoma on Prostate Biopsies', *The American journal of surgical pathology*, 40(12): 1686-92. | <10 pathologists |
| 87 | Samuelson, M. I., S. J. Chen, S. A. Boukhar, E. M. Schnieders, M. L. Walhof, A. M. Bellizzi, R. A. Robinson, and A. Rajan. 2021. 'Rapid Validation of Whole-Slide Imaging for Primary Histopathology Diagnosis', *American Journal of Clinical Pathology*, 155(5): 638-48. | <10 pathologists |
| 88 | Sasaki, H., M. Kido, K. Miki, M. Aoki, H. Takahashi, T. Dokiya, H. Yamanaka, M. Fukushima, and S. Egawa. 2015. 'Results of central pathology review of prostatic biopsies in a contemporary series from a phase III, multicenter, randomized controlled trial (SHIP0804)', *Pathology International*, 65(4): 177-82. | <10 pathologists |
| 89 | Sauter, G., S. Steurer, T. S. Clauditz, T. Krech, C. Wittmer, F. Lutz, M. Lennartz, T. Janssen, N. Hakimi, R. Simon, M. Von Petersdorff-Campen, F. Jacobsen, K. Von Loga, W. Wilczak, S. Minner, M. C. Tsourlakis, V. Chirico, A. Haese, H. Heinzer, B. Beyer, M. Graefen, U. Michl, G. Salomon, T. Steuber, L. H. Budaus, E. Hekeler, J. Malsy-Mink, S. Kutzera, C. Fraune, C. Gobel, H. Huland, and T. Schlomm. 2016. 'Clinical utility of quantitative gleason grading in prostate biopsies and prostatectomy specimens', *European Urology*, 69(4): 592-98. | Not a reproducibility study |
| 90 | Seipel, A. H., B. Delahunt, H. Samaratunga, M. Amin, J. Barton, D. M. Berney, A. Billis, L. Cheng, E. Comperat, A. Evans, S. W. Fine, D. Grignon, P. A. Humphrey, C. Magi-Galluzzi, R. Montironi, I. Sesterhenn, J. R. Srigley, K. Trpkov, T. van der Kwast, M. Varma, M. Zhou, A. Ahmad, S. Moss, and L. Egevad. 2014. 'Diagnostic criteria for ductal adenocarcinoma of the prostate: Interobserver variability among 20 expert uropathologists', *Histopathology*, 65(2): 216-27. | Does not use Gleason scoring |
| 91 | Serbanescu, M. S., C. N. Oancea, C. T. Streba, I. E. Plesea, D. Pirici, L. Streba, and R. M. Plesea. 2020. 'Agreement of two pre-trained deep-learning neural networks built with transfer learning with six pathologists on 6000 patches of prostate cancer from Gleason2019 Challenge', *Romanian journal of morphology and embryology = Revue roumaine de morphologie et embryologie*, 61(2): 513-19. | <10 pathologists |
| 92 | Shah, R. B., J. Li, L. Cheng, L. Egevad, F. M. Deng, S. W. Fine, L. P. Kunju, J. Melamed, R. Mehra, A. O. Osunkoya, G. P. Paner, S. S. Shen, T. Tsuzuki, K. Trpkov, W. Tian, X. J. Yang, and M. Zhou. 2015. 'Diagnosis of Gleason pattern 5 prostate adenocarcinoma on core needle biopsy: an interobserver reproducibility study among urologic pathologists', *The American journal of surgical pathology*, 39(9): 1242-49. | Reproducibility of Gleason pattern 5 only |
| 93 | Shah, R. B., G. Leandro, G. Romerocaces, J. Bentley, J. Yoon, S. Mendrinos, Y. Tadros, W. Tian, and R. Lash. 2016. 'Improvement of diagnostic agreement among pathologists in resolving an "atypical glands suspicious for cancer" diagnosis in prostate biopsies using a novel "Disease-Focused Diagnostic Review" quality improvement process', *Human Pathology*, 56: 155-62. | <10 pathologists |
| 94 | Shah, R. B., Q. Cai, M. Aron, D. M. Berney, J. C. Cheville, F. M. Deng, J. Epstein, S. W. Fine, E. M. Genega, M. S. Hirsch, P. A. Humphrey, J. Gordetsky, G. Kristiansen, L. P. Kunju, C. Magi-Galluzzi, N. Gupta, G. J. Netto, A. O. Osunkoya, B. D. Robinson, K. Trpkov, L. D. True, P. Troncoso, M. Varma, T. Wheeler, S. R. Williamson, A. Wu, and M. Zhou. 2021. 'Diagnosis of "cribriform" prostatic adenocarcinoma: An interobserver reproducibility study among urologic pathologists with recommendations', *American Journal of Cancer Research*, 11(8): 3990-4001. | At high risk of bias, lack of sample representativeness |
| 95 | Silva-Rodriguez, J., A. Colomer, J. Dolz, and V. Naranjo. 2021. 'Self-Learning for Weakly Supervised Gleason Grading of Local Patterns', *IEEE J Biomed Health Inform*, 25: 3094-104. | Not a reproducibility study |
| 96 | Singh, R. V., S. R. Agashe, A. V. Gosavi, and K. R. Sulhyan. 2011. 'Interobserver reproducibility of Gleason grading of prostatic adenocarcinoma among general pathologists', *Indian Journal of Cancer*, 48(4): 488-95. | At high risk of bias, lack of sample representativeness |
| 97 | Skjorten, F. J., A. Berner, S. Harvei, T. E. Robsahm, and S. Tretli. 1997. 'Prostatic intraepithelial neoplasia in surgical resections: Relationship to coexistent adenocarcinoma and atypical adenomatous hyperplasia of the prostate', *Cancer*, 79(6): 1172-79. | <10 pathologists |
| 98 | Sooriakumaran, P., D. P. Lovell, A. Henderson, P. Denham, S. E. M. Langley, and R. W. Laing. 2005. 'Gleason scoring varies among pathologists and this affects clinical risk in patients with prostate cancer', *Clinical Oncology*, 17(8): 655-58. | <10 pathologists |
| 99 | Svanholm, H., H. Starklint, H. Barlebo, and S. Olsen. 1989. 'Histological evaluation of prostatic cancer. 1. Reproducibility of tumour type', *Apmis*, 97(8): 699-704. | <10 pathologists |
| 100 | Svanholm, H., H. Starklint, H. Barlebo, and S. Olsen. 1990. 'Histological evaluation of prostatic cancer (III): Reproducibility of assessment of tumour volume and its possible significance for prognosis', *Apmis*, 98(3): 237-43. | Reproducibility of tumour volume rather than diagnostic categories |
| 101 | Svanholm, H., H. Starklint, H. Barlebo, and S. Olsen. 1990. 'Histological evaluation of prostatic cancer (II): Reproducibility of a histological grading system', *Apmis*, 98(3): 229-36. | Does not use Gleason scoring |
| 102 | Thomsen, F. B. 2015. 'Active surveillance strategy for patients with localised prostate cancer: criteria for progression', *Dan Med J*, 62. | Not a reproducibility study |
| 103 | Torres, R., E. Olson, R. Homer, D. T. Martin, M. J. Levene, S. Perincheri, P. C. Sprenkle, and P. A. Humphrey. 2021. 'Initial Evaluation of Rapid, Direct-to-Digital Prostate Biopsy Pathology', *Archives of pathology & laboratory medicine*, 145(5): 583-91. | <10 pathologists |
| 104 | Truesdale, M. D., P. J. Cheetham, A. T. Turk, S. Sartori, G. W. Hruby, E. P. Dinneen, M. C. Benson, and K. K. Badani. 2011. 'Gleason score concordance on biopsy-confirmed prostate cancer: Is pathological re-evaluation necessary prior to radical prostatectomy?', *BJU International*, 107(5): 749-54. | <10 pathologists |
| 105 | Van Der Kwast, T. H., A. Evans, G. Lockwood, D. Tkachuk, D. G. Bostwick, J. I. Epstein, P. A. Humphrey, R. Montironi, G. J. L. H. Van Leenders, C. G. Pihl, I. Neetens, P. M. Kujala, M. Laurila, C. Mazerolles, L. Bubendorf, A. Finelli, K. Watson, and J. Srigley. 2010. 'Variability in diagnostic opinion among pathologists for single small atypical foci in prostate biopsies', *American Journal of Surgical Pathology*, 34(2): 169-77. | Does not use Gleason scoring |
| 106 | Van Der Kwast, T. H., G. J. Van Leenders, D. M. Berney, B. Delahunt, A. J. Evans, K. A. Iczkowski, J. K. McKenney, J. Y. Ro, H. Samaratunga, J. R. Srigley, T. Tsuzuki, M. Varma, T. M. Wheeler, and L. Egevad. 2021. 'ISUP Consensus Definition of Cribriform Pattern Prostate Cancer', *American Journal of Surgical Pathology*, 45(8): 1118-26. | At high risk of bias, lack of sample representativeness |
| 107 | van der Slot, M. A., E. Hollemans, M. A. den Bakker, R. Hoedemaeker, M. Kliffen, L. M. Budel, N. N. T. Goemaere, and Gjlh van Leenders. 2021. 'Inter-observer variability of cribriform architecture and percent Gleason pattern 4 in prostate cancer: relation to clinical outcome', *Virchows Arch*, 478: 249-56. | <10 pathologists |
| 108 | Veloso, S. G., M. F. Lima, P. G. Salles, C. K. Berenstein, J. D. Scalon, and E. A. Bambirra. 2007. 'Interobserver agreement of Gleason score and modified Gleason score in needle biopsy and in surgical specimen of prostate cancer', *International Braz J Urol*, 33(5): 639-46. | <10 pathologists |
| 109 | Wright, K. C., J. Melia, S. Moss, D. M. Berney, D. Coleman, and P. Harnden. 2011. 'Measuring interobserver variation in a pathology EQA scheme using weighted kappa for multiple readers', *Journal of Clinical Pathology*, 64(12): 1128-31. | At high risk of bias, lack of sample representativeness |
| 110 | Yatani, R., T. Soga, S. Miura, K. Itoh, H. Nakano, H. Nakabayashi, I. Kusano, T. Shiraishi, M. Noda, and T. Yoshide. 1986. 'Observer variability in the histopathologic grading of prostatic carcinoma. [Japanese]', *Gan No Rinsho*, Japan journal of cancer clinics. 32(2): 176-80. | Non-English manuscript |
| 111 | Zelic, R., F. Giunchi, L. Lianas, C. Mascia, G. Zanetti, O. Andren, J. Fridfeldt, J. Carlsson, S. Davidsson, L. Molinaro, P. H. Vincent, L. Richiardi, O. Akre, M. Fiorentino, and A. Pettersson. 2021. 'Interchangeability of light and virtual microscopy for histopathological evaluation of prostate cancer', *Scientific reports*, 11(1): 3257. | <10 pathologists |
| 112 | Zhou, M., J. Li, L. Cheng, L. Egevad, F. M. Deng, L. P. Kunju, C. Magi-Galluzzi, J. Melamed, R. Mehra, S. Mendrinos, A. O. Osunkoya, G. Paner, S. S. Shen, T. Tsuzuki, K. Trpkov, W. Tian, X. Yang, and R. B. Shah. 2015. 'Diagnosis of "Poorly Formed Glands" Gleason Pattern 4 Prostatic Adenocarcinoma on Needle Biopsy: An Interobserver Reproducibility Study Among Urologic Pathologists With Recommendations', *The American journal of surgical pathology*, 39(10): 1331-39. | At high risk of bias, lack of sample representativeness |

**Table S3d: Reasons for records excluded from diagnostic drift studies (n=24).**

| No. | Article | Reasons for exclusion |
| --- | --- | --- |
| 1 | Abouelfadel, Z., G. J. Miller, L. M. Glode, B. Akduman, R. E. Donohue, A. Nedrow, and E. D. Crawford. 2002. 'High Gleason scores and lower prostate-specific antigen levels in a single institution over the past decade', *Clinical Prostate Cancer*, 1(2): 115-17. | Not a diagnostic drift study |
| 2 | Bass, E. J., C. Orczyk, A. Grey, A. Freeman, C. Jameson, S. Punwani, N. Ramachandran, C. Allen, M. Emberton, and H. U. Ahmed. 2019. 'Targeted biopsy of the prostate: does this result in improvement in detection of high-grade cancer or the occurrence of the Will Rogers phenomenon?', *BJU International*, 124(4): 643-48. | Not a diagnostic drift study |
| 3 | Boehm, K., H. Borgmann, T. Ebert, T. Höfner, E. Khaljani, M. Schmid, W. Schulze-Seemann, P. Weib, and J. Herden. 2021. 'Stage and Grade Migration in Prostate Cancer Treated With Radical Prostatectomy in a Large German Multicenter Cohort', *Clin Genitourin Cancer*, 19: 162-66.e1. | Not a diagnostic drift study |
| 4 | Bollito, E., C. Terrone, A. Volpe, F. Porpiglia, C. Cracco, M. Poggio, S. Grande, L. Righi, M. Bellina, M. Papotti, and R. M. Scarpa. 2008. 'Changes in prostate cancer at radical prostatectomy during the prostate specific antigen era: an Italian experience', *Anal Quant Cytol Histol*, 30: 152-9. | Not a diagnostic drift study |
| 5 | Cao, Y., W. Zhang, Y. Li, J. Fu, H. Li, X. Li, X. Gao, K. Zhang, and S. Liu. 2021. 'Rates and trends in stage-specific prostate cancer incidence by age and race/ethnicity, 2000-2017', *Prostate*, 81(14): 1071-77. | Not a diagnostic drift study |
| 6 | Connor, M. J., M. Winkler, and H. U. Ahmed. 2020. 'Survival in Oligometastatic Prostate Cancer-A New Dawn or the Will Rogers Phenomenon?', *JAMA Oncol*, 6: 185-86. | Not a diagnostic drift study |
| 7 | Danneman, D., L. Drevin, D. Robinson, P. Stattin, and L. Egevad. 2015. 'Gleason inflation 1998-2011: a registry study of 97,168 men', *BJU Int*, 115: 248-55. | Not a diagnostic drift study |
| 8 | Garg, H., A. Seth, P. Singh, and R. Kumar. 2021. 'Changing trends in robot-assisted radical prostatectomy: Inverse stage migration-A retrospective analysis', *Prostate International.* | Not a diagnostic drift study |
| 9 | Gilliland, F., T. M. Becker, A. Smith, C. R. Key, and J. M. Samet. 1994. 'Trends in prostate cancer incidence and mortality in New Mexico are consistent with an increase in effective screening', *Cancer Epidemiol Biomarkers Prev*, 3: 105-11. | Not a diagnostic drift study |
| 10 | Gomella, L. G. 1998. 'The Will Rogers phenomenon in prostate cancer: a good thing', *Cancer J Sci Am*, 4: 19-21. | Commentary of Vijayakumar article |
| 11 | Han, M., A. W. Partin, S. Piantadosi, J. I. Epstein, and P. C. Walsh. 2001. 'Era specific biochemical recurrence-free survival following radical prostatectomy for clinically localized prostate cancer', *J Urol*, 166: 416-9. | Not a diagnostic drift study |
| 12 | Han, M., A. W. Partin, D. Y. Chan, and P. C. Walsh. 2004. 'An evaluation of the decreasing incidence of positive surgical margins in a large retropubic prostatectomy series', *J Urol*, 171: 23-6. | Not a diagnostic drift study |
| 13 | Javali, T. D., P. M. Dogra, N. P. Gupta, P. Singh, P. Chatterjee, and A. K. Dinda. 2013. 'Diagnostic dilemma in histopathology report following robot assisted laparoscopic prostatectomy: tumour 'hide and seek'', *Indian J Cancer*, 50: 170-4. | Not a diagnostic drift study |
| 14 | Jhaveri, F. M., E. A. Klein, P. A. Kupelian, C. Zippe, and H. S. Levin. 1999. 'Declining rates of extracapsular extension after radical prostatectomy: evidence for continued stage migration', *J Clin Oncol*, 17: 3167-72. | Not a diagnostic drift study |
| 15 | Master, V. A., T. Chi, J. P. Simko, V. Weinberg, and P. R. Carroll. 2005. 'The independent impact of extended pattern biopsy on prostate cancer stage migration', *J Urol*, 174: 1789-93; discussion 93. | Not a diagnostic drift study |
| 16 | McGuire, B. B., B. Anglim, S. Loeb, B. T. Helfand, R. Grainger, R. Flynn, T. McDermott, Q. Hu, P. R. Cooper, A. Fennell, W. J. Catalona, and J. Thornhill. 2014. 'Radical prostatectomy outcomes during prostate-specific antigen era in Ireland compared to a matched American population', *Journal of Clinical Urology*, 7(3): 170-75. | Not a diagnostic drift study |
| 17 | Mian, B. M., D. J. Lehr, C. K. Moore, H. A. Fisher, R. P. Kaufman, Jr., J. S. Ross, T. A. Jennings, and T. Nazeer. 2006. 'Role of prostate biopsy schemes in accurate prediction of Gleason scores', *Urology*, 67: 379-83. | Not a diagnostic drift study |
| 18 | Noldus, J., M. Graefen, P. Hammerer, R. P. Henke, and H. Huland. 1998. 'Development of tumor selection on the basis of pathological staging in clinically localized prostate carcinoma. [German]', *Urologe - Ausgabe A*, 37(2): 195-98. | Article in German |
| 19 | Sheng, I. Y., W. Wei, Y. W. Chen, T. D. Gilligan, P. C. Barata, M. C. Ornstein, B. I. Rini, and J. A. Garcia. 2021. 'Implications of the United States Preventive Services Task Force Recommendations on Prostate Cancer Stage Migration', *Clin Genitourin Cancer*, 19: e12-e16. | Not a diagnostic drift study |
| 20 | Thangarasu, M., S. P. Jayaprakash, N. Selvaraj, S. Bafna, R. Paul, C. Mahesh, N. Jain, A. Balakrishnan, and A. Sivaraman. 2021. 'A prospective study on the efficacy of cognitive targeted transrectal ultrasound prostate biopsy in diagnosing clinically significant prostate cancer', *Research and Reports in Urology*, 13: 207-13. | Not a diagnostic drift study |
| 21 | Vijayakumar, S., F. Vaida, R. Weichselbaum, and S. Hellman. 1998. 'Race and the Will Rogers phenomenon in prostate cancer', *Cancer J Sci Am*, 4: 27-34. | Not a diagnostic drift study |
| 22 | Washington, S. L., M. Bonham, J. M. Whitson, J. E. Cowan, and P. R. Carroll. 2012. 'Transrectal ultrasonography-guided biopsy does not reliably identify dominant cancer location in men with low-risk prostate cancer', *BJU Int*, 110: 50-5. | Not a diagnostic drift study |
| 23 | Winkler, M. H., F. A. Khan, I. M. Hoh, A. A. Okeke, M. Sugiono, P. McInerney, G. B. Boustead, R. Persad, A. V. Kaisary, and D. A. Gillatt. 2004. 'Time trends in case selection, stage and prostate-specific antigen recurrence after radical prostatectomy: a multicentre audit', *BJU Int*, 93: 725-9. | Not a diagnostic drift study |
| 24 | Wurnschimmel, C., M. Kachanov, M. Wenzel, P. Mandel, P. I. Karakiewicz, T. Maurer, T. Steuber, D. Tilki, M. Graefen, and L. Budaus. 2021. 'Twenty-year trends in prostate cancer stage and grade migration in a large contemporary german radical prostatectomy cohort', *Prostate*, 81(12): 849-56. | Not a diagnostic drift study |

**SUPPLEMENT 4: Complete risk of bias assessment.**

Low overall risk of bias: low risk of bias for all domains.

Moderate overall risk of bias: moderate/unknown risk of bias in 1 domain.

High overall risk of bias: high risk of bias in 1 or more domains or moderate/unknown risk of bias in 2 or more domains.

**Figure S4a:** Complete risk of bias assessment for the 4 active surveillance studies


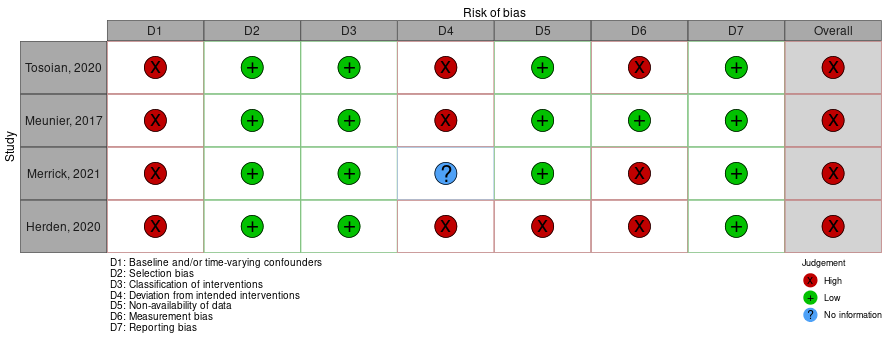


**Figure S4b:** Complete risk of bias assessment for the 4 autopsy studies

**
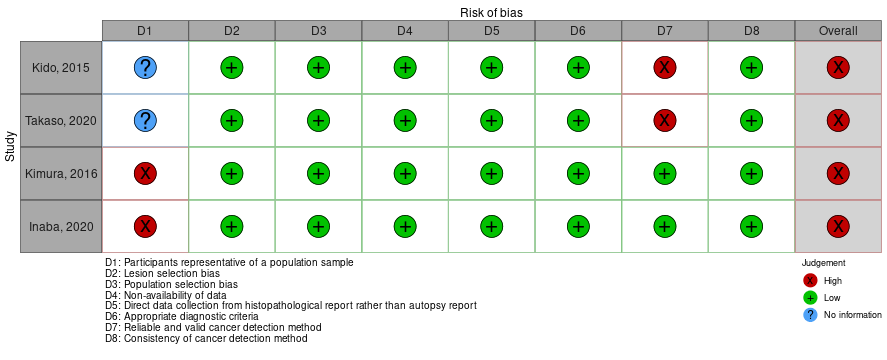
**

**Figure S4c:** Complete risk of bias assessment for the 8 reproducibility studies


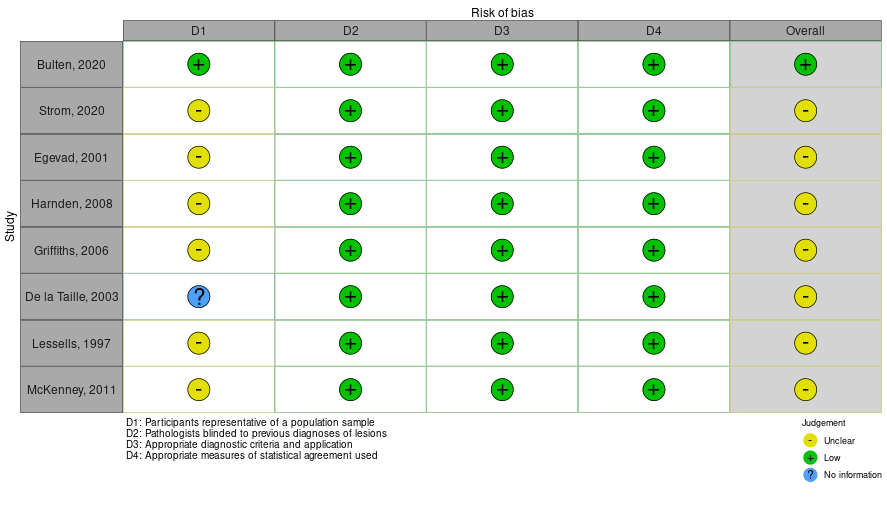


**Figure S4d:** Complete risk of bias assessment for the 4 diagnostic drift studies

**
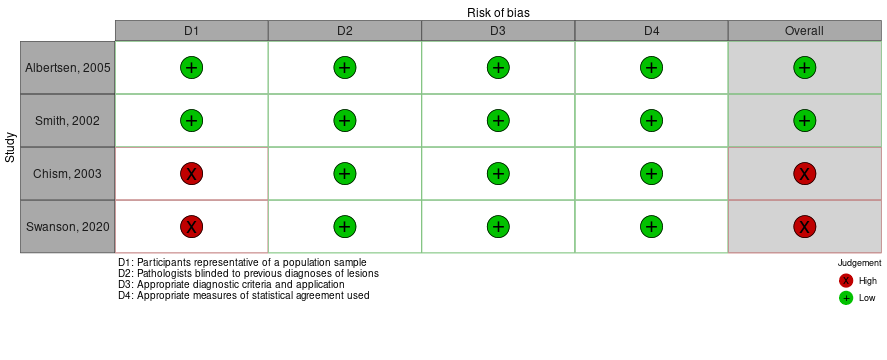
**
